# Engaging an HIV vaccine target through the acquisition of low B cell affinity

**DOI:** 10.1038/s41467-023-40918-2

**Published:** 2023-08-28

**Authors:** Larance Ronsard, Ashraf S. Yousif, Faez Amokrane Nait Mohamed, Jared Feldman, Vintus Okonkwo, Caitlin McCarthy, Julia Schnabel, Timothy Caradonna, Ralston M. Barnes, Daniel Rohrer, Nils Lonberg, Aaron Schmidt, Daniel Lingwood

**Affiliations:** 1 The Ragon Institute of Mass General, The Massachusetts Institute of Technology and Harvard University, 400 Technology Square, Cambridge, MA 02139 USA; 2 Bristol-Myers Squibb, 700 Bay Rd, Redwood City, CA 94063-2478 USA; 3 Department of Microbiology, Harvard Medical School, Boston, MA 02115 USA

**Keywords:** Somatic hypermutation, Vaccines, Antibodies, HIV infections

## Abstract

Low affinity is common for germline B cell receptors (BCR) seeding development of broadly neutralizing antibodies (bnAbs) that engage hypervariable viruses, including HIV. Antibody affinity selection is also non-homogenizing, insuring the survival of low affinity B cell clones. To explore whether this provides a natural window for expanding human B cell lineages against conserved vaccine targets, we deploy transgenic mice mimicking human antibody diversity and somatic hypermutation (SHM) and immunize with simple monomeric HIV glycoprotein envelope immunogens. We report an immunization regimen that focuses B cell memory upon the conserved CD4 binding site (CD4bs) through both conventional affinity maturation and reproducible expansion of low affinity BCR clones with public patterns in SHM. In the latter instance, SHM facilitates target acquisition by decreasing binding strength. This suggests that permissive B cell selection enables the discovery of antibody epitopes, in this case an HIV bnAb site.

## Abstract (web summary)

Broadly neutralizing antibodies (bnAbs) for HIV have been difficult to elicit with one issue being the low B cell affinity required. Here the authors use a transgenic mouse bearing human-like antibody repertoires to show that low affinity B cells persist which enables vaccine expansion of antibodies against the CD4 binding site, a conserved HIV bnAb target.

## Introduction

Engaging structurally diverse antigens is a hallmark of antibodies. This is achieved by two sequential diversification steps that shape their antigen-binding sites. Paratopic diversity is generated by V(D)J recombination during B cell development^1,2^ and additional diversity is acquired by somatic hypermutation (SHM) when B cells enter germinal centers (GCs) and undergo affinity maturation^3,4^. Affinity selection was once thought to constrain clonal diversity to a more limited number of high affinity BCRs. However, methodological improvements for measuring GC clonal composition during the immune response reveal that such ‘winner-take-all’ events are relatively rare, and that as yet undefined stochastic factors unrelated to BCR affinity can strongly influence selection, enabling less stringent competition and the longer-term survival of low affinity B cell clones^5–8^. Permissiveness in B cell selection has also been explored by chronically immunizing BCR transgenic mice with non-cognate antigen, a procedure that eventually results in the elicitation of affinity matured cognate antibodies^9^.

Permissive B cell selection is also suggestive of a ‘natural window’ for expanding humoral responses that are specifically seeded by low affinity B cell antigen interactions, most notably, human broadly neutralizing antibodies (bnAbs) against HIV and influenza virus^10–17^. In these scenarios, weak/non-detectable germline antibody affinity for cognate antigen may be consequence of structural constraints within the target epitopes^12,18–20^ and has been viewed as one factor that contributes to the immunological subdominance of bnAbs^10,11,14–16,21–23^. Germline stimulating vaccine concepts have sought to address this by engineering enhanced immunogen affinity to the bnAb precursor, so as to preferentially activate human bnAb development pathways^23–26^. In the HIV space, efforts have focused on triggering the development of VRC01-class bnAb lineages, which mature from low/non detectable affinity B cells and which block infection by mimicking the functionally conserved CD4 binding site (CD4bs) of the virus^12,20,21,24,27–45^. VRC01-class bnAbs are unconventional since these paratopes center around the germline-encoded antigen-binding CDRH2 loop provided by the human antibody V_H_ gene IGVH1-2*02^12,13,20^. By contrast, the antigen binding sites of most antibodies are formed by the hypervariable CDRH3 loops, which are centrally positioned, stochastically generated, and are unique to each B cell clone^46^. Such CDRH3-dominant antibody paratopes can also engage the CD4bs to provide HIV neutralization potency and breadth that is comparable to VRC01-class bnAbs, a feature achieved by maturation of rare human B cell lineages that can also be seeded by low affinity B cells^12^.

In this work, we evaluate a prediction of permissive B cell affinity selection: that the persistence of low affinity BCRs provides a natural window for human antibodies to engage the affinity-constraining CD4bs on HIV. To test this, we deploy transgenic mice recapitulating human-like antibody CDRH3 diversity^47–52^ and immunize with simple monomeric HIV glycoprotein envelope (Env) antigens to elicit humoral immunity. Here we show that responses targeting the HIV CD4bs are achieved by conventional affinity maturation but also via decreases in antibody affinity. Permissiveness as a mechanism that naturally supports B cell discovery of antibody epitopes and conserved vaccine targets is discussed.

## Results

### Enhanced elicitation of D368R-sensitive antibodies following sequential immunization of humanized mice with heterologous strain-variant HIV Env monomers

**Fig. 1:**
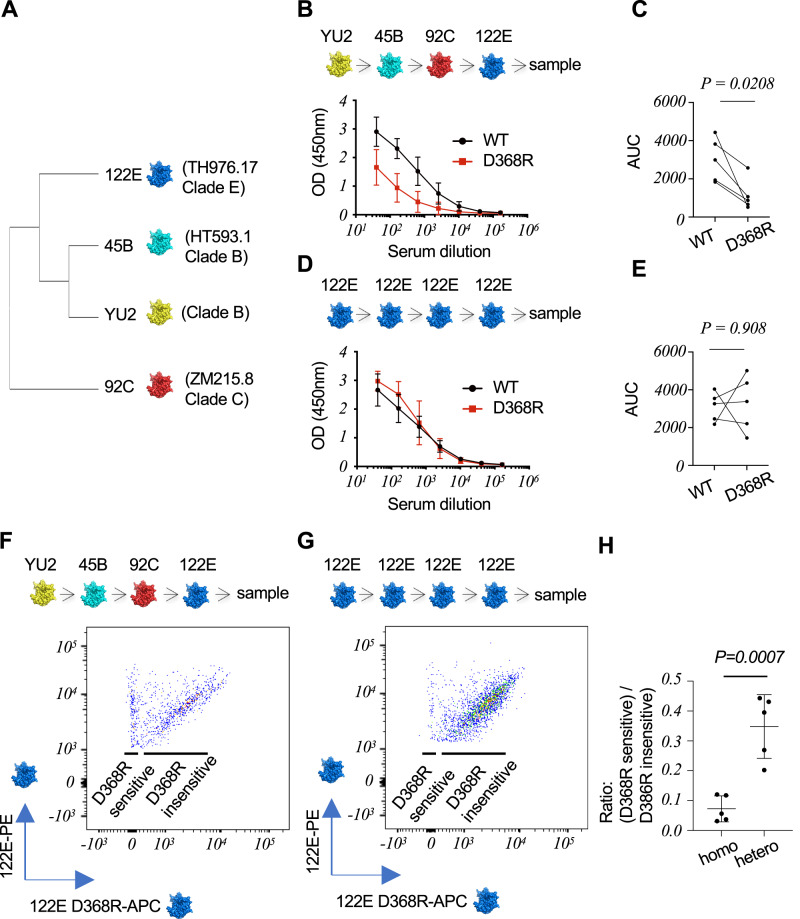
Vaccine-elicitation of D386R-sensitive antibodies targeting the CD4bs in humanized mice. **A** Phylogenetically distinct HIV Env monomers deployed in this study (strains TH976.17, HT593.1, YU2 and ZM215.8, referred to as: 122E, 45B, YU2, and 92 C, respectively; see also Supplementary Fig. 1A). **B** Serum antibodies elicited in IGHV1-2 HC2 mice following sequential immunization with heterologous Env (mean ± SD, serum antibody reactivity against WT vs D368R forms of 122E Env, n = 5 biologically independent animals). **C** Corresponding serum antibody reactivity against WT vs D368R forms of 122E Env as compared by area under the curve (AUC, *n* = 5 biologically independent animals, *P* = 0.0208, two-sided paired *T* test of ratio, see also Supplementary Fig. 2 for serum reactivity to the other Env strains). **D** Serum antibodies elicited in IGHV1-2 mice following sequential immunization with homologous 122E Env (mean ± SD, serum antibody reactivity against WT vs D368R forms of 122E Env, *n* = 5 biologically independent animals). **E** Corresponding serum antibody reactivity against WT vs D368R forms of 122E Env as compared by AUC (*n* = 5 biologically independent animals, *P* = 0.908, two-sided paired *T* test of ratio). **F** D386R-sensitive versus D368R-insensitive 122E specific memory IgG B cells expanded following immunization with heterologous Env (gated on: CD3^-^/CD19^+^/IgM^-^/IgD^-^/IgG^+^/GL7^-^/CD38^+^/122E Env-PE+/122E Env-APC-Cy7^+^, see Supplementary Fig. 3 for full gating; see also Supplementary Fig. 5 for memory BCR reactivity to the other Env sequences used in the vaccine regimen). **G** D386R-sensitive versus D368R-insensitive Env specific memory IgG B cells expanded following immunization with homologous Env (gated on: CD3^-^/CD19^+^/IgM^-^/IgD^-^/IgG^+^/GL7^-^/CD38^+^/122E Env-PE+/122E Env-APC-Cy7^+^). **H** Ratio of D368R-sensitive to D368R-insensitive IgG B cells expanded by the homologous (homo) versus heterologous (hetero) vaccination regimens (mean ± SD, *n* = 5 biologically independent animals, *P* = 0.0007, two-sided Student’s *T* test; see also Supplementary Fig. 4 for comparison to sequential immunization with a cocktail of the four Env antigens). Data are derived from two independent immunization experiments (Experiment 1 = (**B**–**E**); Experiment 2 = (**F**–**H**) where mice were pre-bled and sequentially immunized through the intraperitoneal route with individual Env antigens at weeks 0, 3, 6 and 9. Blood was collected two weeks after each immunization and then three weeks after the final inoculation. Spleen was also collected at this final time point.

Env gp120 monomers predominantly elicit antibody responses against non-neutralizing and non-conserved epitopes^53–55^. By contrast, sequential immunization of mice with sequence-variant Env monomers can enable selective boosting of B cell memory upon conserved features, including the CD4bs^56,57^. We first screened different sequential immunization regimens with established strain-variant Env monomers^12,58^ to define an inoculation sequence that recapitulated this phenomenon in wildtype (WT) C57Bl/6 mice (Supplementary Fig. 1) and then within IGHV1-2 HC2 mice (vaccination sequence: YU2 - > 45B - > 92 C - > 122E) (Fig. 1A–E). The integrated HC2 locus, provides for expression of near-normal and unrestricted human-like CDRH3 diversity, usage of single human V_H_ genes (in this case IGHV1-2*02) and WT C57BL/6-comparable levels of SHM and affinity maturation^47–52^. Serum IgG focusing to the CD4bs supersite was functionally defined by reactivity to Env but not Env-D368R, a well described CD4bs binding mutant^59–62^. D368R sensitivity does not encompass all CD4bs-directed antibodies, and because YU2 is also more favorable to binding by CD4bs mAbs^60^, we deployed a set of mutations established to reduce this more promiscuous recognition (D368R + A281R + G366R + P369R)^12^. Antibodies against the CD4bs of the other Env strains (45B, 92 C, 122E) was defined by D368R-sensitivity only. We found that the order of the Env antigens was important for eliciting CD4bs antibodies, and the titer of these antibodies was enhanced in IGHV1-2 HC2 mice relative to WT C57Bl/6 (Fig. 1A–C, Supplementary Figs. 1, 2). Moreover, we observed a time lag in boosting antibody titers during the heterologous immunization regimen (prime ~ post-boost <post boost 2<post boost 3; Supplementary Fig. 2), consistent with introducing antigenic distance between Env strains^57^. Applying sequential immunization with strain variant Env to refocus the antibody response upon a single conserved feature, such as the CD4bs, has been less clear when trimeric gp140 Env immunogens are deployed in this way^63,64^. Consistent with expectations^56,57^, sequential (4x) immunization regimens deploying inoculum with homologous 122E Env monomer alone [the last Env antigen of the heterologous vaccine regimen (YU2 - > 45B - > 92 C - > 122E)] did not reproducibly elicit the same titer 122E D368R-sensitive serum IgG after the final immunization step (Fig. 1D, E).

To track the vaccine responses at the B cell level, we applied Env-PE/Env-APC-Cy7 and Env-D368R-APC as B cell flow cytometry probes to identify antigen specific class switched memory B cells^51,65^ expanded (Fig. 1F–H, Supplementary Figs. 3–5). D368R-sensitive IgG memory B cells were defined as CD3^-^/CD19^+^/IgM^-^/IgD^-^/IgG^+^/GL7^-^/CD38^+^/Env-PE^+^/Env-APC-Cy7^+^/Env-D368R-APC^-^, whereas D368R-insensitive memory B cells were CD3^-^/CD19^+^/IgM^-^/IgD^-^/IgG^+^/ GL7^-^/CD38^+^/Env-PE^+^/Env-APC-Cy7^+^/Env-D368R (D368R + A281R + G366R + P369R in the case of YU2^12^)-APC^+^ (Supplementary Fig. 3). We compared 4x sequential immunization with homologous Env (122E - > 122E - > 122E - > 122E) versus heterologous Env (YU2 - > 45B - > 92 C - > 122E), in all cases using 122E Env sequence as the final immunization step and antigen probe [122E -PE^+^/122E -APC-Cy7^+^/122E -D368R^-^ (D368R-sensitive) vs 122E -PE^+^/122E -APC-Cy7^+^/122E -D368R^+^ (D368R-insensitive)]. Consistent with expectations^56,57^, the proportion of D368R-sensitive IgG memory B cells was enhanced in the heterologous vaccine sequence (Fig. 1F–H). Also as expected^56,57^, this gain-of function was not observed when the heterologous Env antigens were sequentially immunized as a Env cocktail (YU2 + 45B + 92 C + 122E) (Supplementary Fig. 4).

To more comprehensively define D368R-sensitive IgG memory B cells expanded by the heterologous immunization sequence (YU2 - > 45B - > 92 C - > 122E -> post vax) we evaluated the antigenicity post-vax to the other Env antigens that were immunized at earlier immunization timepoints: (1) YU2-PE^+^/YU2-APC-Cy7^+^/YU2-(D368R + A281R + G366R + P369R)-APC^-^ (mutation-sensitive on YU2) versus YU2-PE^+^/YU2-APC-Cy7^+^/YU2- (D368R + A281R + G366R + P369R)-APC^+^ (mutation-insensitive on YU2); (2) 45B-PE^+^/45B-APC-Cy7^+^/45B-D368R-APC^-^ (D368R-sensitive on 45B) versus 45B-PE^+^/45B-APC-Cy7^+^/45B-D368R-APC^+^ (D368R-insensitive on 45B); and (3) 92C-PE^+^/92C-APC-Cy7^+^/92C-D368R-APC^-^ (D368R-sensitive on 92 C) versus 92C-PE^+^/92C-APC-Cy7^+^/92C-D368R-APC^+^ (D368R-insensitive on 92 C). We note that after the 4x immunization regimen, a higher proportion of D368R-sensitivity versus D368R-insensitivity was observed on the earlier versus later Env antigens (YU2 > 45B > 92 C > 122E) (Supplementary Fig. 5). This suggests that while D368R sensitivity on memory B cells does not completely overlap across the divergent Env sequences, D368R-sensitivity memory (and not D368R-insensitivity) from earlier antigens was retained and/or boosted as new heterologous immunogens were successively applied. There was also a trend towards higher MFI for double positive (mutation-insensitive) B cells expanded for the earlier immunized Env strains (Supplementary Fig. 5) suggesting that higher affinity ‘off-target’ clones may also accompany the response.

### Public D368R-sensitive antibody clones bearing high and non-detectable affinities for cognate antigen are reproducibly expanded by the vaccine

Compared with the serum antibodies expanded by homologous immunization (122E - > 122E - > 122E - > 122E) the serum antibodies elicited by the heterologous immunization regimen (YU2 - > 45B - > 92 C - > 122E) bound the final immunogen 122E with similar IgG titer but lowered strength, as measured by reduced resistance to chaotropic urea^66,67^ (Supplementary Fig. 6A, B). We confirmed that affinity maturation was functional in IGHV1-2 HC2 mice and comparable to WT C57Bl/6 animals since immunization with NP-ovalbumin elicited serum antibodies with similar NP2/NP23 ratios, which increased at the same rate after immune challenge (Supplementary Fig. 6C, D). NP2 and NP23 are ELISA reagents that display sparse and more densely spaced NP on BSA. An increase in serum antibody reactivity to sparse vs densely spaced NP on BSA is an index of affinity maturation within the primary antibody response to NP (as elicited by a non-BSA carrier protein displaying NP)^68,69^. Collectively, these results indicated that IGHV1-2 mice had WT C57Bl/6-like affinity maturation, but that the enhanced titer of D368R-sensitive antibodies elicited by the heterologous immunization regimen was also potentially supported by lower binding affinity.

**Fig. 2:**
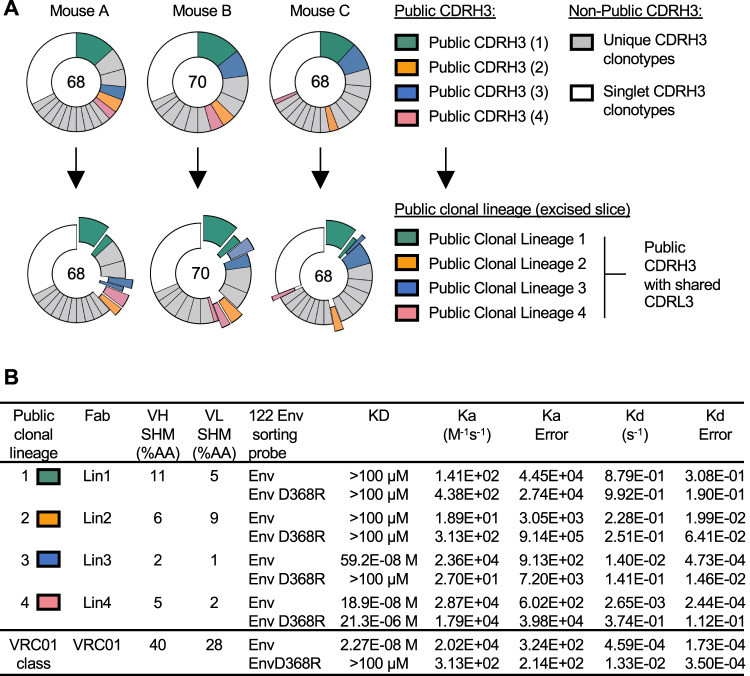
Expansion of public IGHV1-2 BCR lineages with both affinity matured and non-detectable binding underscore the elicitation of D368R sensitive antibodies. Single D368R-sensitive memory IgG B cells (CD19^+^/IgD^-^/IgM^-^/IgG^+^/ GL7^-^/CD38^+^/122E-PE^+^/122E-APC-Cy7^+^/122E D368R-APC^-^) from animals receiving the heterologous immunization regimen (see also Fig. 1F, Supplementary Figs. 3–5) were sorted from *n* = 3 biologically independent IGHV1-2 HC2 animals. **A** Sequencing the memory B cells yielded 68 BCRs in Mouse A, 70 BCRs in Mouse B, and 68 BCRs in Mouse C (see also Supplementary Data 1). Public B cell pathways were first assigned based on clonotype (usage of a shared CDRH3 sequence) using Cloanalyst software^52,70–77^. These public B cell pathways were then further subdivided into public clonal lineages (public CDRH3 + shared CDRL3) revealing four clonal lineages that were reproducibly expanded in all the mice (see also Supplementary Data 1). **B** Fabs from each public clonal lineage recombinantly and then tested for affinity to original sorting probes (122E Env or 122E Env-D368R). Binding was evaluated using bilayer interferometry (BLI). The equilibrium dissociation constant (KD) values were calculated by applying a 1:1 binding isotherm using vendor-supplied software. KD values above 100 µM are beyond the limit of detection for this instrument^17^. The low affinity Lin1 and Lin2 BCR sequences chosen for this analysis contain a conserved pattern of SHM that was independently reproduced within each public clonal lineage within each mouse (Supplementary Figs. 7, 8). Data are from one vaccination experiment that yielded antigen specific IgG memory BCRs in the three mice; details of each individual B cell clone are provided in Supplementary Data 1.

To define this at the antigen receptor level, we applied FACS and single cell sequencing to the D368R-sensitive memory IgG BCRs elicited by the heterologous immunization regimen within *n* = 3 vaccine recipients (CD3^-^/CD19^+^/IgM^-^/IgD^-^/IgG^+^/GL7^-^/CD38^+^/122E-PE^+^/122E-APC-Cy7^+^/122E-D368R-APC^-^; *n* = 68, 70, and 68 BCRs from each animal) (Fig. 2A, Supplementary Fig. 3, Supplementary Data 1). In these experiments, 122E-PE, 122E-APC-Cy7 and 122E-D368R-APC served as the sorting probes since 122E Env was the final immunogen within the immunization sequence (YU2 - > 45B - > 92 C - > 122E). Using Cloanalyst software^52,70–77^, we first identified public B cell lineages by CDRH3 sequences that were shared across the vaccine recipients (Fig. 2A, Supplementary Data 1). We further subdivided the public lineages into public B cell clones (BCRs using public CDRH3 + CDRL3 in each of the animals) (Fig. 2A, Supplementary Data 1). VRC01-class antibodies (defined by IGHV1-2*02 usage and the short five amino acid CDRL3 signature deployed by this antibody type^20^) were not expanded, however four public clonal lineages (Lin1, Lin2, Lin3, and Lin4) revealed reproducible expansion of BCRs with a range of affinities for the 122 Env sorting probe: nanomolar (Lin3 and Lin4) to non-detectable (>100 uM) (Lineages Lin1 and Lin2) (Fig. 2B, Supplementary Data 1). Somatic hypermutation (SHM) occurred in all of the public clones (2-11% in V_H_, and 1-9% in V_L_) (Fig. 2B). In addition to being public B cell clones, the low affinity Lin1 and Lin2 BCR sequences chosen for analysis contain a core pattern of HC mutations that were reproducibly yet independently expanded within each mouse (Supplementary Figs. 7, 8).

### SHM achieves D368R sensitivity via the acquisition of low affinity (B cell Lineage 1)

The identification of public B cell clones with strong and weak affinity for Env suggested that vaccine-expansion of BCRs bearing D368R sensitivity is supported by non-homogenous affinity selection. To evaluate whether the weak affinity clones (Public B cell Lineages 1 and 2) support CD4bs targeting at >100 µM, we reconstituted the Ig sequences as monospecific BCRs within a Ig-negative reporter B cell line^78^. Surface display of Ig in this system can resolve low-affinity antibody-antigens beyond what is detectable with purified components and is also routinely deployed in antigen receptor triggering studies^10,12,47,78–87^.

**Fig. 3:**
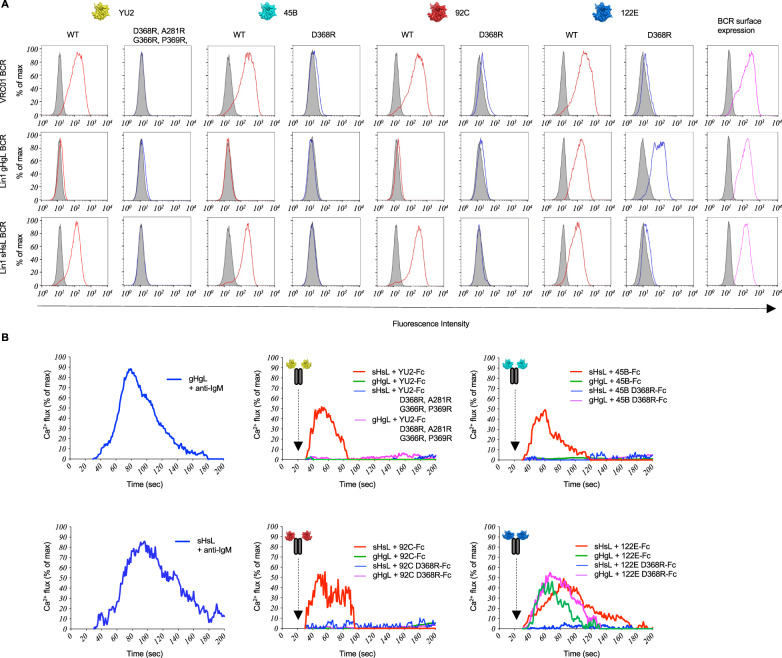
BCR antigenicity and signaling within Public Clonal Lineage 1 (Lin1). **A** sHsL and gHgL BCRs in Lineage 1, along with VRC01 BCRs were expressed in our B cell reporter system^78^ and evaluated by flow cytometry for binding to fluorescent versions of WT Env (red lines) vs D368R Env (blue lines; D368R + A281R + G366R + P369R in the case of YU2^12^) for each antigen used in the immunization regimen. Gray histograms represent binding to BCR isotype control or binding to surface BCR negative for LC expression. **B** Acquisition of D368R sensitivity, as further resolved by gHgL BCR vs sHsL BCR triggering in response to bivalent Env-Fc, Env-D368R-Fc or anti-IgM as a positive control. All Env variants used in the heterologous immunization regimen were tested. Presented is the Ca^2+^ flux activity, measured kinetically by the ratiometric Ca^2+^ sensing dye fura red and normalized to total flux capacity, as defined by the ionophore ionomycin^78^. (**A**) vs (**B**) represents two independent experiments to evaluate BCR antigen recognition via the two orthogonal methods (binding to membrane presented BCR and BCR triggering following antigen exposure). Antigenicity of sHsL vs gHgL BCR was independently confirmed by reversing the fluorescent label on Env vs Env-D368R (Supplementary Fig. 9), and LC vs HC contribution to the acquisition of D368R sensitivity was evaluated by independently comparing antigenicity of sHgL vs sHsL BCR (Supplementary Fig. 10). BCR triggering was first established in response to 122E Env-Fc vs 122E Env-D368R-Fc (Supplementary Fig. 11), and then independently evaluated for Fc-presentation of all the Env strains used in the heterologous immunization regimen, as shown in this Figure (**B**).

**Fig. 4:**
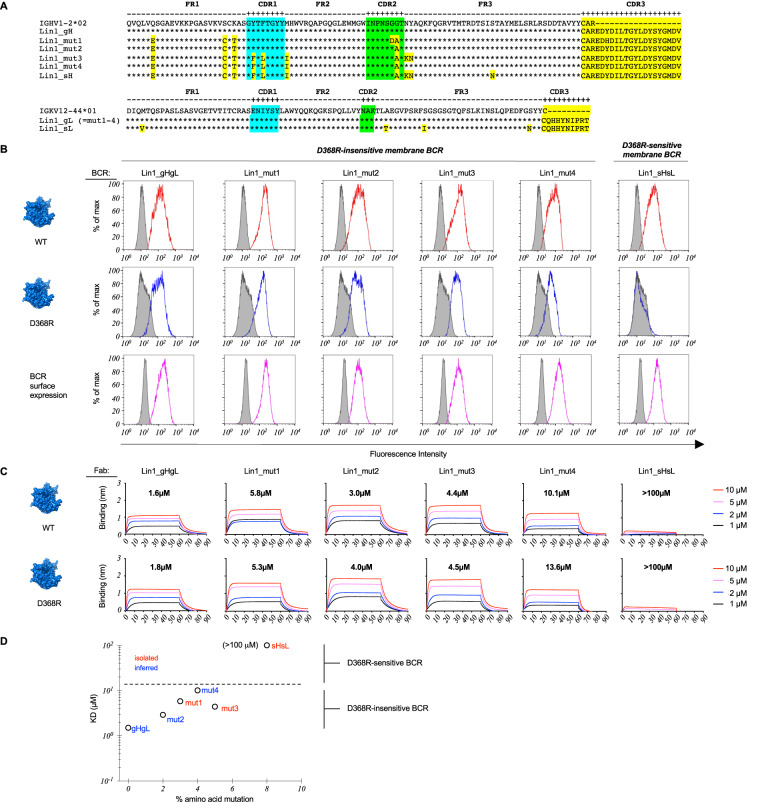
Scanning the antigen surface through SHM and low affinity in Public B cell Lineage 1 (Lin1). **A** sHsL, gHgL and inferred intermediate sequences within B cell Lineage 1 (Lin1_mut1-4). Lin 1 is public clonal lineage that also uses a public pattern of SHM that is conserved across vaccine recipients (Supplementary Figs. 7, 12). **B** sHsL, gHgL and intermediate BCR sequences were expressed as IgM BCRs in a B cell reporter system^78^ and evaluated by flow cytometry for binding to fluorescent versions of WT Env (red lines) vs D368R Env (blue lines). Gray histograms represent binding to BCR isotype control or binding to surface BCR negative for LC expression. Data presented represents one experiment. **C** sHsL, gHgL and Lin1_mut1-4 expressed as Fabs and then evaluated for binding to Env vs Env-D368R using bilayer interferometry (BLI). The equilibrium dissociation constant (KD) values were calculated by applying a 1:1 binding isotherm using vendor-supplied software (see also Supplementary Table 1). KD values above 100 µM are beyond the limit of detection for this instrument^17^. Data presented represents one experiment. **D** Permissiveness in B cell affinity selection as a window for scanning the antigen surface through SHM. Acquisition of D368R sensitivity in the membrane BCR format (see **B**) is underscored by lower affinity for cognate antigen, as measured in the Fab format (see **C**). **B**, **C** represent independent experiments to compare BCR recognition by two orthogonal methods (binding by membrane presented BCR versus monomeric antibody affinity). A phylogenetic tree denoting the positions of sHsL, gHgL and intermediates is presented in Supplementary Fig. 12.

For Lin1, we evaluated BCR as mature (sHsL), V_H_V_L_ gene reverted (gHgL), and inferred mutation steps (Figs. 3, 4, Supplementary Figs. 7, 9–12). We found that membrane presented sHsL BCR engaged all of the Env strain variants but not their D368R forms, similar to VRC01 BCR, a human HIV bnAb that engages the conserved CD4bs (Fig. 3A, Supplementary Fig. 9). By contrast, gHgL BCR bound only to the 122E Env variant, and this occurred without sensitivity to D368R (Fig. 3A, Supplementary Fig. 9). Both Lin1 sHsL and Lin1 sHgL BCRs were D368R-sensitive, indicating that mutations in HC (and not LC) were primarily responsible for sensitivity to this mutation (Supplementary Fig. 10).

We then tested whether sHsL vs gHgL BCR antigenicity was reflected in BCR triggering, as measured by Ca^2+^ fluxing. We first evaluated receptor triggering in response to bivalent forms of 122E (122E-Fc vs 122E-D368R-Fc; Supplementary Fig. 11), then in response to Fc forms for all of the Env strains (Fig. 3B). All Env-Fc strains triggered sHsL BCR singling and there was no response to all strains of Env-D368R (Fig. 3B, Supplementary Fig. 11). By contrast, gHgL BCR only signaled in response to the 122E strain, and was triggered by both 122E-Fc and 122E-D368R (Fig. 3B, Supplementary Fig. 11). This suggested that Public Clonal Lineage 1 was triggered by 122E Env, and that only after SHM was D368R sensitivity acquired.

We next defined the relationship between BCR D368R sensitivity, Fab affinity in solution, and SHM within Lin1 using inferred intermediates from the public pattern of SHM in this B cell lineage that was reproduced across vaccine recipients (Fig. 4A–D, Supplementary Figs. 7, 12, Supplementary Table 1). We found that SHM could change formally D368R-insensitive membrane BCRs to become sensitive, consistent with ‘antigen scanning’, and that the acquisition of D368R-sensitivity was associated with a drop in affinity for cognate antigen (Fig. 4C, D, Supplementary Table 1).

### SHM achieves D368R sensitivity via acquisition of low affinity (B cell Lineage 2)

**Fig. 5:**
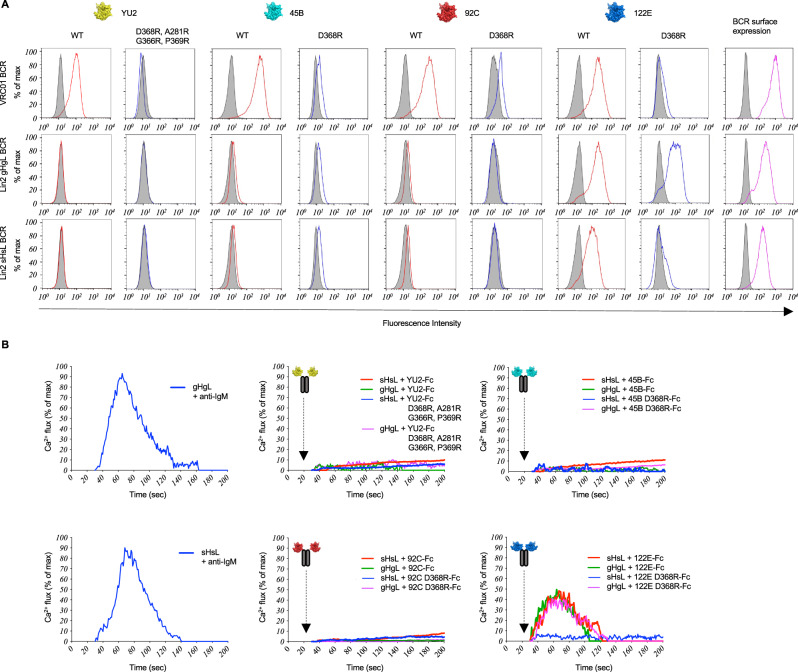
BCR antigenicity and signaling within Public Clonal Lineage 2 (Lin2). **A** sHsL and gHgL BCRs in Lineage 2, along with VRC01 BCRs were expressed in our B cell reporter system^78^ and evaluated by flow cytometry for binding to fluorescent versions of WT Env (red lines) vs D368R Env (blue lines; D368R + A281R + G366R + P369R in the case of YU2^12^) for each antigen used in the immunization regimen. Gray histograms represent binding to BCR isotype control. Data presented represents one experiment. **B** Acquisition of D368R sensitivity, was further resolved by gHgL BCR vs sHsL BCR triggering in response to bivalent Env-Fc, Env-D368R-Fc or anti-IgM as a positive control. All Env variants used in the heterologous immunization regimen were tested. Presented is the Ca^2+^ flux activity, measured kinetically by the ratiometric Ca^2+^ sensing dye fura red and normalized to total flux capacity, as defined by the ionophore ionomycin. Data presented represents one experiment. (**A**) vs (**B**) represents two independent experiments to evaluate BCR antigen recognition via the two orthogonal methods (binding to membrane presented BCR and BCR triggering following antigen exposure). Antigenicity of sHsL vs gHgL BCR was also independently confirmed by reversing the fluorescent label on Env vs Env-D368R (Supplementary Fig. 13), and LC vs HC contribution to the acquisition of D368R sensitivity was independently evaluated by comparing antigenicity of sHgL vs sHsL BCR (Supplementary Fig. 14). Additionally, BCR triggering was first established in response to 122E Env-Fc vs 122E Env-D368R-Fc (Supplementary Fig. 15), and then independently evaluated for Fc-presentation of all the Env strains used in the heterologous immunization regimen, as shown in this Figure (**B**).

**Fig. 6:**
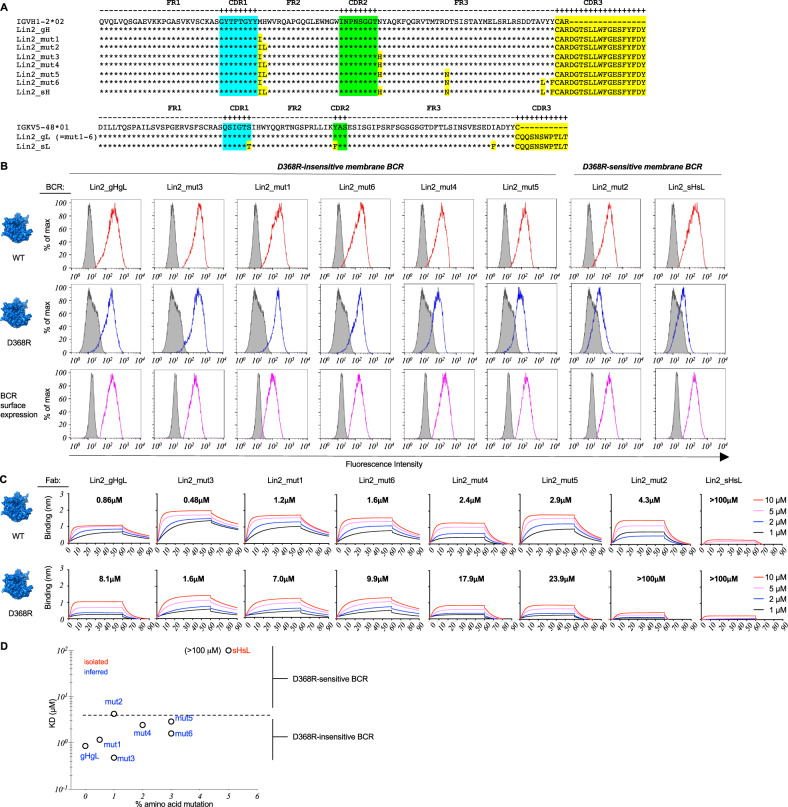
Scanning the antigen surface through SHM and low affinity in Public Clonal Lineage 2 (Lin2). **A** sHsL, gHgL and inferred intermediate sequences within B cell Lineage 2 (Lin2_mut1-6). Lin 2 is public clonal lineage that also uses a public pattern of SHM that is conserved across vaccine recipients (Supplementary Figs. 8, 16). **B** sHsL, gHgL and intermediates were evaluated by flow cytometry for binding to WT Env (red lines) vs D368R Env (blue lines) in our B cell reporter system^78^. Gray histograms depict binding to isotope control or binding to surface BCR negative for LC expression. Data presented represents one experiment. **C** sHsL, gHgL and Lin2_mut1-6 expressed as Fabs and then evaluated for binding to Env vs Env-D368R using BLI. The equilibrium dissociation constant (KD) values were calculated by applying a 1:1 binding isotherm using vendor-supplied software (see also Supplementary Table 1). KD values above 100 µM are beyond the limit of detection for this instrument^17^. Data presented represents one experiment. **D** Permissiveness in B cell affinity selection as a window for scanning the antigen surface through SHM. Acquisition of D368R sensitivity in the membrane BCR format (see **B**) is underscored by lower affinity for cognate antigen, as measured in the Fab format (see **C**). **B**, **C** represent independent experiments to compare BCR recognition by two orthogonal methods (binding by membrane presented BCR versus monomeric antibody affinity). A phylogenetic tree denoting the positions of sHsL, gHgL and intermediates is presented in Supplementary Fig. 16.

In Public Clonal Lineage 1, antibody affinity for Env was inversely related to D368R sensitivity, consistent with antigen scanning through permissive B cell selection. To confirm that this was a general property, we applied the same methodology to the other public D368R-sensitive BCR clonal lineage that were expanded with non-detectable affinity (>100 µM) to cognate antigen (Lin2). As before, we used BCR antigenicity and signaling in the B cell reporter system to first resolve low-affinity membrane BCR target specificity and signaling^10,78^ (Figs. 5, 6, Supplementary Figs. 8, 13–16), and then measured affinities of the corresponding Fabs to quantify binding strength, if detectable in solution (Fig. 6C, Supplementary Table 1).

We found that sHsL BCR of Public Clonal Lineage 2 also engaged 122E with D368R-sensitivity and gHgL BCR bound both WT and D368R forms of this Env (Fig. 5A, B, Supplementary Fig. 13). As with Lin1, the acquisition of D386R-sensitivity was HC dominant since both Lin2 sHsL and Lin2 sHgL were D368R-sensitive (Supplementary Fig. 14). However, sHsL BCR did not engage the other Env antigens, indicative of differences within the D368R-sensitive epitope patch engaged by Lin1 versus Lin2. We confirmed this antigenicity and acquisition of D368R-sensitivity in Lin2 by antigen receptor triggering, where sHsL BCR was activated by 122E-Fc only, whereas gHgL BCR was triggered by both 122E-Fc and 122E-D368R-Fc (Fig. 5B, Supplementary Fig. 15). As with the BCR antigenicity, sHsL and gHgL BCRs were not responsive to the other Env strains.

Analogous to Lin1, we note that SHM enabled the transition from a D368R-insensitive to D368R-sensitive membrane BCR (Fig. 6A–D, Supplementary Table 1). As we incorporated the inferred mutation steps [from the public pattern of mutations seen across vaccine recipients (Supplementary Figs. 8, 16)], D368R sensitivity in the membrane BCRs was underscored by a greater decrease in Fab affinity for Env-D368R relative to Env, but the lineage was also marked by an overall loss in affinity for both Env and Env-D368R as the mutations were applied (Fig. 6C, D, Supplementary Table 1). Notably, a single amino acid change (H35L) in mut2 conferred D368R sensitivity and concomitant lower affinity, a targeting feature that could be gained, lost and regained, consistent with a pathway for antigen scanning via SHM (Fig. 6D).

### One-step restricted accumulation of affinity during sequential immunization in mice

**Fig. 7:**
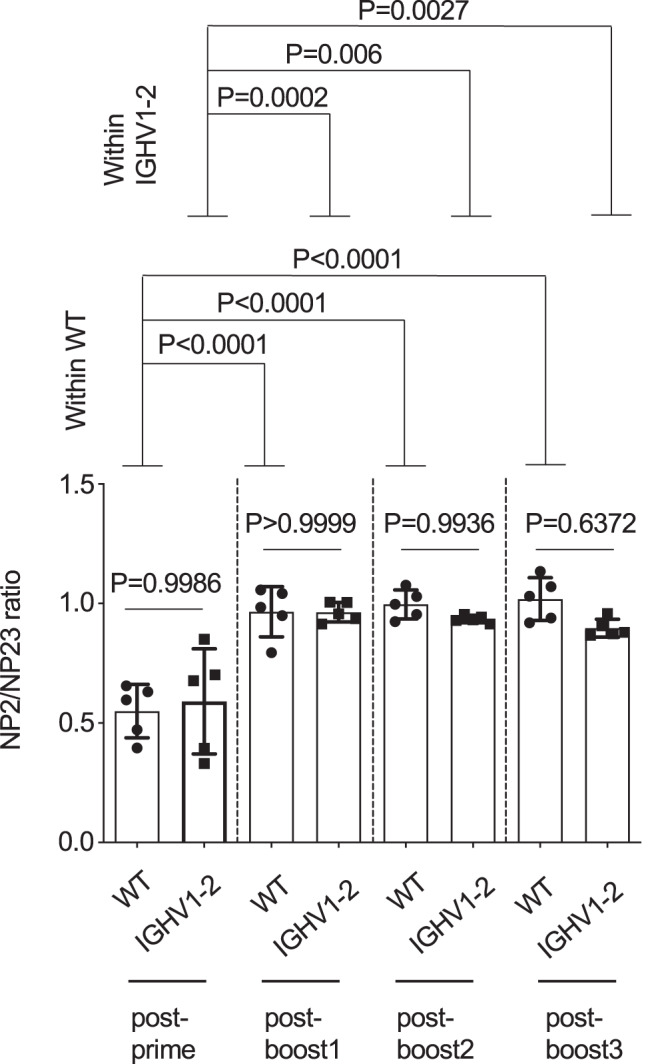
Asymptotic/one-step restricted accumulation of affinity following sequential immunization in mice. WT C57Bl/6 and IGHV1-2 HC2 mice were sequentially immunized with NP-ovalbumin (4x) at weeks: 0, 3, 6 and 9 and ratio of NP2 to N23 binding by the serum IgG response was measured two weeks after each immunization [mean ± SD], *n* = 5 biologically independent animals per genotype, two-factor ANOVA. Factor 1= genotype; Factor 2 = immunization number. Following identification of an immunization number effect (*P* < 0.0001) but not genotype effect (*P* = 0.8238), factor 2 was further analyzed with Tukey’s test. The *P* values for the resultant pairwise comparisons are presented in the figure. Data are from one vaccination experiment.

The GCs formed following sequential immunization in mice are, by large majority, populated by germline B cells and not memory B cells^88,89^. The dominance of germline B cells in GC reactions after each antigen exposure could account for germline triggering and maturation of Public Clonal Lineages 1 and 2 after the last immunization step in the regimen. This organization predicts an asymptotic or single step-restricted accumulation of affinity in B cell memory during sequential immunization. To test this, we re-performed our 4x sequential immunization regimen in WT C57Bl/6 and IGHV1-2 HC2 mice except now deploying NP-ovalbumin. We found that in both genotypes, NP-2/NP-23 ratios were restricted to a one-step increase which then remained as a non-accumulative maximum throughout the immunization regimen (Fig. 7). This suggests that the memory B cells responsible for the secondary antibody response (memory recall) are, on average, constrained to a single round of affinity maturation.

## Discussion

Affinity selection within GCs can be artificially homogenized by engineering antigen with high affinity for cognate BCR, as in the case of germline stimulating vaccines^21,31,32,40^. However, there is also functional activity to permissive B cell selection, as demonstrated by immunizing BCR transgenic mice with non-cognate antigen^9^. In these experiments, immunization eventually led to antigen complementarity through secondary diversification by SHM^9^. Our data suggests that this principle also enables vaccine-expansion of B cells targeting sites that constrain germline antibody affinity, a hallmark of some human bnAbs^10–17,42,43^.

We note that antibody-focusing to the conserved CD4bs on HIV was partially underscored by expansion of public D368R sensitive B cell lineages and SHM patterns associated with lowered affinity. This weak affinity (KD > 100 µM) is analogous to the permissive GC B cell clones previously shown to expand at non-detectable antigen affinity following immunization in WT mice^5^. Membrane presentation of BCR can resolve antibody-antigen interactions that are below the detection level of conventional binding assays^10,78^, and while caveats of V_H_ and V_L_ gene reversion to explore germline BCR antigen specificities may exist, we applied membrane presentation of immunoglobulin as a tool to detect the acquisition of D368R sensitivity at low affinity.

Analogous epitope-shifting through reduction in antigen affinity has been reported during clonal redemption of autoreactive GC B cells^90^. Likewise, longer lived germinal centers have recently been demonstrated to become populated by B cell “invaders” bearing low and/or non-detectable affinity to cognate antigen^91,92^. Permissive B cell selection (and epitope discovery therein) would appear to maximize BCR repertoire diversity, even during on-going immune reactions.

Our regimen did not expand the IGHV1-2*02-dependent VRC01-class antibodies, but rather appeared to support low affinity engagement through the ‘default’ CDRH3-dominant mode of antigen complementarity. In humans, CDRH3-dominant bnAbs often deploy longer chain lengths that can access the glycan-shielded protein surface on HIV Env^93^. The mouse antibody repertoire averages ~10–12 amino acids^94^, but the D368R-sensitive BCRs expanded had longer human-like CDRH3s (public CDRH3 length= 20 ± 1.63 amino acids; non-public CDRH3 length = 17 ± 3.40 amino acids, see Supplementary Data 1). This was facilitated by the HC2 locus, which endows for a length distribution that is more similar to humans (average 15-16 amino acids)^47,48,51^. The importance of a longer human-like CDRH3 repertoire may also explain the elicitation of higher titer D368R-sensitive serum antibodies after applying the same immunization regimen in HC2 versus WT C57Bl/6 mice.

While elicitation of CD4bs antibodies is not uncommon in HIV infection or vaccine studies, the threshold for generating broad CD4bs-dependent neutralization remains high^60–62,95,96^. In this context, permissiveness in B cell selection may provide a natural window for seeding CD4bs directed bnAbs, as these antibodies often develop from germline BCRs with low/non-detectable affinity for antigen^12,13,42,43^. We could also expect permissive low affinity BCRs to serve as substrate for further SHM and conventional affinity selection. Conceivably, this could be experimentally probed by titrating up and down the 122E dose in presence or absence of an Env competitor that displays the CD4bs with different antigenicity and potentially different affinity (e.g. YU2). Polyreactivity can also mark CD4bs-directed bnAbs^97^, and this could also support downstream affinity selection since polyreactivity itself is correlated with heightened affinity for viral targets^98^.

The practical feasibility of vaccine-expanding low affinity clones through rational vaccine design remains uncertain, both in relation to competition with other B cell clonotypes and where memory B cells can have limited re-entry into GCs^88,89^. Limited GC-re-entry by memory B cells is also supported by our demonstration of an affinity limit which underscores repeat antigen exposure in mice. Slow antigen delivery/release strategies may be required to address these obsticles^99,100^.

While the stochastic factors responsible for permissive B cell selection are not known, we provide evidence that permissiveness enables SHM to further scan the antigenic surface without B cell elimination due to low affinity. Our results indicate that this operates in tandem with conventional affinity maturation, providing flexibility to commit and un-commit B cell lineages to specific epitopes. Ultimately this secures a baseline ability to engage an antibody target that constrains germline contact affinity.

## Methods

### Ethics statement

The research conducted complies with ethical regulations and biosafety approved by institutional committees at Mass General Brigham (MGB Institutional Biosafety Committee, protocol # 2014B000035; MGB Institutional Animal Care and Use Committee, protocol # 2014N000252). This research does not include human participants.

### Animals

Wildtype C57Bl/6 mice were obtained from Jackson labs. The HC2 mice are an established transgenic model in which human V_H_ gene usage is constrained to user-defined gene segments, while allowing for recombination with diverse human D and J segments, generating an antibody CDRH3 repertoire that is similar to humans and which supports affinity matured antibodies following immunization with protein antigens^47–52^. HC2 mice using the human V_H_ gene IGHV1-2*02 were deployed in which LC input was from the mouse repertoire^47,50–52^. The HC2 mice were a gift to D.L. from Bristol-Myers Squibb (Redwood City, CA) and their use requires an MTA with BMS. The animals were maintained within Ragon Institute’s HPPF barrier facility. IGHV1-2 HC2 breeders were maintained as heterozygotes backcrossed to WT C57Bl/6 J mice **(**IGHV1-2 HC2^+/–^ / C57Bl/6 J IgH^+/-^)^47–52^. Both male and female animals were used at 8 weeks of age. The light cycles in the animal room were set on a 12 h light cycle [7AM-7PM (ON) 7PM-7AM (OFF)]. The temperature range for the room was 68–73 degrees Fahrenheit and the humidity index was from 30 to 70%. At the end of experiments, the animals were euthanized by CO_2_ inhalation (30% of the chamber volume/min).

### Recombinant antigens and B cell probes

Recombinant proteins were constructed from synthetic DNA molecules cloned into the mammalian expression plasmid pVRC8400^78^. Untagged gp120 Env core antigens (strains TH976.17, HT593.1, YU2 and ZM215.8; herein referred to as: 122E, 45B, YU2, and 92 C, respectively) along with their D368R variants (D368R + A281R + G366R + P369R in the case of YU2^12^) were purified on a 17B column^12,13,58^. The sequences of these Env monomers and their CD4bs mutant forms are provided in Supplementary Data 1. Here 293F cells were grown in Freestyle media (Life Technologies) and transfected with 500 μg/L of each Env construct (293fectin Reagent, Life Technologies, Cat # 12347019). At day five, the culture was centrifuged (2000 × *g*, 10 min) and the supernatant was filtered (VacuCap 0.2 μm filters, Fisher Scientific, Cat # 501979599) loaded onto Protein A-agarose resin that had been covalently pre-coupled to the CD4i antibody 17B (see below for generation of the 17B column). This resin was washed (6 column volumes of PBS) and then the bound gp120 was eluted directly into 50 mM Tris, pH 8, using 1.5 column volumes of low pH IgG elution buffer (Pierce, Cat # 21004). The gp120 proteins were then concentrated using Amicon Ultra concentrators (30 kDa MWCO, Millipore, Cat # UFC8030) and were further separated by size exclusion FPLC using a Superdex 200 Increase 10/300 column (Cytiva, Cat # 289990944).

To generate the 17B affinity column, 17B HC and LC plasmids (kindly provided by James Robinson, Tulane University), were first co-expressed in the 293 F system and then harvested on day 6 and affinity purified on Protein G-Agarose (Thermo Scientific, Cat # 20398) using the same low pH elution step described above. The 17B was then further separated by size exclusion FPLC [Superdex 200 Increase 10/300 column (Cytiva, Cat # 289990944)]. Ten milligrams of the purified 17B was added to 5 mL of Protein A-agarose resin [Thermo Scientific, Cat # 20365] that had been resuspended in antibody binding buffer (50 mM sodium borate, pH 8.2, Sigma, Cat # 08059). Following 1 h incubation at room temperature, the column was washed in antibody binding buffer (5–10 mL increments) and the 17B was then immobilized via addition of disuccinimidyl suberate (DSS) (Pierce Cat # 21555) at 6.5 mg DSS per 1 mL of Protein A. After one hour at room temperature, the column was washed with 5 column volumes of PBS and the remaining non-reacted NHS-ester groups were blocked by addition of 0.1 M ethanolamine (Sigma, Cat # E9508) pH 8.2 in diethyl pyrocarbonate (Sigma, Cat # D5758) at 1 mL per mL of column volume. The resin was then washed with 5 column volumes of low pH IgG elution buffer and the 17B was stored in PBS containing 0.02% sodium azide (Sigma, Cat # S2002).

To generate fluorescent B cell probes of the gp120 antigens, avi-tagged versions of these proteins (WT and D368R versions; D368R + A281R + G366R + P369R in the case of YU2^12^) were expressed and purified using the 17B affinity column as described above and then biotinylated using a commercial biotin ligase kit according to the manufacturer’s instructions (Avidity, BirA-500). Recombinant influenza hemaggluitinin (HA) (avi-tagged Y98F HA from A/New Caledonia/20/1999) was also generated and biotinylated as an additional ligand control^47,51,78^. The biotinylated proteins were then conjugated with PE-streptavidin (eBioscience, Cat# 12-431787), APC-Cy7-streptavidin (Biolegend, Cat # 405208), or APC-streptavidin (Biolegend, Cat # 405207) through iterative complex formation as we have described before^78^. Here the fluorescent SA conjugates were added to biotinylated gp120 in five increments, such that the final molar ratio of probe to streptavidin label was 4:1 to saturate the label. After each stepwise addition of fluorescent label, the mixture was incubated for 20 min and set rotating at 4 °C.

### Immunization regimens

Mice were pre-bled and sequentially immunized through the intraperitoneal route with individual Env antigens at weeks 0, 3, 6 and 9. Blood or spleen was collected two weeks after the first three immunizations and then three weeks after the final inoculation. The spleen was also obtained three weeks after the final immunization step to evaluate the antigen specific IgG B cell lineages expanded. For homologous and heterologous immunization regimens, each inoculum consisted of 15 µg of Env in a 100ul volume containing 50% w/v Sigma Adjuvant System (Cat # S6322)^47,51,79,101^. In some experiments, all four Env antigens were sequentially immunized as a cocktail with 15ug of total Env retained in the inoculum. In some experiments, 15 µg of NP-ovalbumn (Biosearch Technologies, Cat # N-5051-10) was deployed as the antigen.

### ELISA

gp120 antigens (WT and D368R versions; D368R + A281R + G366R + P369R in the case of YU2^12^) and NP coupled to BSA at high and low density (NP-23 and NP-2, respectively; Biosearch, custom NP loading), were coated onto 96 well Nunc MaxiSorp plates (Life Technologies, Cat # 44240421) at 200 ng per well. The plates were then blocked with 3% milk in PBS for 1 h, washed with PBS, and then incubated with immune sera, which were first diluted in PBS at 1:40 and then serial diluted at 1:4. Serial dilutions of the monoclonal antibody VRC01 were also made at 1:4 in PBS. After 1 h, the plates were washed with PBS and 0.05% Tween 20 (PBST) and incubated with anti-IgG-HRP (GE Healthcare, Cat # NA931) at 1:5000 dilution in PBS. The plates were washed with PBST and then developed using tetramethylbenzidine substrate. The developer reaction was quenched with 1 N sulfuric acid (Fisher Scientific, Cat # 7664-93-9), and read at 450 nm using a microplate absorbance reader (Teacan Infinite m1000 Pro Mannedorf, Switzerland).

The polyclonal binding strength of the serum antibodies elicited after immunization with gp120 antigens, was evaluated by a urea resistance ELISA assay^66,67,102^. In this assay, Env antigen coating, blocking, and serial dilutions of the immune sera were as above, however after incubating with immune sera, the wells were washed three times with PBST and then once with 7 M urea (Sigma, Cat # 57-13-6) (dissolved in PBS) or PBS as a control. After incubation for 15 min at room temperature, the wells were washed three times with PBST and incubation with anti-IgG-HRP (GE Healthcare, Cat # NA931) and subsequent development, quenching and absorbance reading occurred as described above.

### B cell flow cytometry and sorting

Mouse spleens were gently ground in PBS, lysed in 1xACK lysis buffer (Lonza, Cat # BP10-548E) and filtered through a 70 µm cell strainer. The cells were washed (PBS) and then stained with Aqua Live/Dead amine-reactive dye (ThermoFisher, Cat # L34957) at 0.025 mg/ml for 2 min. The cells were then washed and then incubated with a 1x cocktail of flow cytometry antibodies (each antibody at a final dilution of 1:100): anti-CD3 Brilliant Violet 785 (Biolegend, Cat # 100232); anti-CD19 BV421 (Biolegend, Cat # 115537), anti-IgM BV605 (Biolegend, Cat # 406523); anti-IgD BUV395 (BD Biosciences, Cat# 564274); anti-IgG PerCPCy5.5 (Cat # 405314); anti-GL7 PE-Cy7 (Cat # 144620); anti-CD38 Alexa 594 (Cat # 102725); along with 0.25 µg of Env-PE, Env-APC-Cy7 and Env-D368R-APC probes [122E-PE, 122E-APC-Cy7, 122E-D368R-APC; or YU2-PE, YU2-APC-Cy7, YU2-D368R-APC; or 45B-PE, 45B-APC-Cy7, 45B-D368R-APC; or 92C-PE, 92C-APC-Cy7, 45B-D368R-APC]. The mixture was incubated at 4 °C for 1 h. The cells were then washed twice, resuspended in PBS and then subjected to FACS where between two and five million events were recorded on a FACSAria Fusion Sorter (BD Biosciences) located within a biosafety cabinet. Three weeks after the final immunization step in the heterologous immunization sequence (YU2 - > 45B - > 92 C - > 122E), single antigen specific memory B cells (D368R-sensitive = CD3^-^/CD19^+^/IgD-/IgM^-^/IgG^+^/GL7^-^/CD38^+^/122E-PE^+^/122E-APC-Cy7^+^/122E-D368R-APC^-^; or D368R-insensitive = CD3^-^/CD19^+^/IgD^-^/IgM^-^/IgG^+^/GL7^-^/CD38^+^/122E-PE^+^/122E-APC-Cy7^+^/122E-D368R-APC^+^) were sorted directly into 96 well plates containing RLT lysis buffer (Qiagen, Cat # 79216) containing 1% beta-mercaptoethanol (Sigma, Cat # M6250). Compensation for antibody staining was performed using AbC Total Compensation Beads (ThermoFisher, Cat # A10497). Downstream analyses of the data were performed using FlowJo software version 10.7.2 (TreeStar).

### BCR sequencing

BCR libraries were enriched from single-cell whole transcriptome amplification (WTA) products that were generated using the Smart-Seq2 protocol^47,103^. Here the final WTA products were subjected to two 0.8x (v/v) SPRI bead-based cleanups and then verified by High Sensitivity D5000 ScreenTape (Agilent Technologies Inc, Cat # 5067-5592) and quantified and normalized using Qubit dsDNA HS Assay kit (Thermofisher, Cat # Q32854). To enrich BCR from the WTA products, the heavy and light chain of each single cell’s BCR (FR1 to CDR3) were amplified separately [HotStarTaq Plus Master Kit, Qiagen, Cat # 203645], using a pool of partially degenerate V region specific gene primers against all possible IGHV (human) or IGLV (mouse) and IGKV (mouse) segments in the FR1 region (final concentration: 10 µM each) and reverse primers against the heavy or light constant regions (final concentration: 10 µM each), and where these primers were also attached to the Illumina P7 (V region) and P5 (constant region) sequences^47^. Following BCR amplification, a 0.8x (v/v) SPRI cleanup was performed, and amplicons were quantified and normalized to 0.2–0.5 ng/µL. We next added cellular barcodes along with Illumina sequencing adapters (based on Nextera XT Index Adapters, Illumina Inc.) to each amplified heavy and light chain using step-out PCR (Kapa HiFi HotStart ReadyMix; Fisher Scientific Cat # 50-196-5217). After purification with a 0.8x (v/v) SPRI and pooling of the HC and LC samples, the paired single-cell BCR libraries were sequenced using paired end 250 × 250 reads and 8 × 8 index reads on an Illumina MiSeq System (MiSeq Reagent Kit v2 (500-cycle), Cat # MS-102-2003). The sequences were then evaluated by pairing HC and LC reads by their cellular barcodes, reconstructing overlapping sequences (PandaSeq^104^), and aligning the reads against the human IMGT database^105^. For the latter step, PCR/sequencing error correction was applied with MigMAP, a wrapper for IgBlast (https://github.com/mikessh/migmap). For each single cell, the consensus V-chain and L/K-chains were determined by combining all reads with the same CDR3 sequence and calling the top heavy and light chain sequences by frequency. HC or LC sequences without at least 25 reads or frequency two-times greater than the next sequence of the same chain was denoted as without consensus.

### B cell lineage analysis

We used the program Cloanalyst to define B cell lineages (shared CDRH3) and also B cell clones (paired CDRH3 + CDRL3 with shared VDJ + VJ origin) from the sequencing data (https://www.bu.edu/computationalimmunology/research/software/). Cloanalyst defines clonotypes based on single heavy chains or light chain nucleotide sequence^52,70–77^. The program uses V and J gene calls, CDR3 length, and a similarity measure of the CDR3 with a threshold that varies depending on how mutated the V gene region is. As the mutation frequency increases for the pair of antibodies, the threshold for CDR3 similarity decreases. We used Cloanalyst to analyze the BCR sequences from D368R-sensitive memory B cells (CD3^-^/CD19^+^/IgM^-^/IgD^-^/IgG^+^/GL7^-^/CD38^+^/Env-PE^+^/Env-APC-Cy7^+^/Env-D368R-APC^-^) isolated from *n* = 3 biologically independent mice (n = 68, 70, and 68 BCRs sequenced from each animal). Public CDRH3 were first defined based on heavy chain clonal relatedness across the three mice. Separately, light chain lineages (shared CDRL3) were analyzed by Cloanalyst. The public clones were then manually identified as those BCRs from a public CDRH3 that also shared a clonally related, paired light chain lineage. Phylogenetic trees to visualize relatedness were generated from the heavy chain nucleotide sequences using the maximum likelihood method with the Tamura-Nei model in MEGA11 software^106,107^. Either Lin1_gH or Lin2_gH was used as the baseline for tree construction.

### Antigen recognition and by vaccine-expanded BCRs

To evaluate antigen recognition in BCR format, the mature (sHsL), variable-region germline reverted (gHgL) and intermediate forms of the vaccine-expanded mAbs were expressed in a BCR-negative reporter Ramos B cell line, engineered to display mono-specific IgM BCRs of interest^78^ (Lin1_gHgL, Lin1_sHsL, Lin1_mut1, Lin1_mut2, Lin1_mut 3, Lin1_mut 4; Lin2_gHgL, Lin2_sHsL, Lin2_mut1, Lin2_mut2, Lin2_mut 3, Lin2_mut 4, Lin2_mut 5, Lin2_mut 6). This reporter system consists of stable expression of ectopic BCR through lenti-viral mediated delivery of membrane anchored HC and LC sequences and has been now widely described and deployed as a tool to rank-order candidate immunogens in antigen receptor triggering studies^12,47,78–85^ and its display of user-defined BCR sequences has also been validated by sequencing^86^.

To assess antigen binding, the BCR reporter cell lines were first cultured in complete RPMI (cRPMI) [RPMI (Gibco, Cat # 12633012) with 15% FBS (Sigma, Cat # F4135) + 1× L-glutamine (Fisher Scientific, Cat # 25030081) + 1× of penicillin + streptomycin (Life Technologies, Cat # 15140122)], washed in PBS, and then incubated with violet fluorescent reactive dye (ThermoFisher; 0.025 mg/ml for 2 min). The cells were washed again in PBS and then incubated for 1 h, 4 °C, in PBS containing 1% FBS with either: 0.25 µg Env-PE; 0.25 µg Env-368R-APC; or PE anti-kappa chain (eBioscience, Cat # 12-9970-42) to monitor BCR surface expression in each line. VRC01 IgM BCR cells^12,86^ served as a positive control and FE53 IgM BCR cells^10,86^ served as a isotype control (except for anti-kappa staining since FE53 also has a kappa LC; in this case, IgM surface negative B cells served the negative control). In some experiments, 0.25 µg Y98F HA-PE and 0.25 µg Y98F HA-APC were applied as control ligands. Cells were washed (2x) and then fixed in 0.5% PFA in PBS. Cell fluorescent intensities were then measured by flow cytometry (LSR II, BD Biosciences). Downstream analyses of the data were performed using FlowJo software version 10.7.2 (TreeStar).

### BCR triggering

BCR signaling in the B cell reporter system requires antigens to be multivalent^10,47,78^. Accordingly, Fc-fusion forms of our gp120 antigens we generated by attaching the Fc region (CH2 and CH3 domains) of the human IgG1 heavy chain and the hinge region to the C-terminus of the Env and Env-D368R (D368R + A281R + G366R + P369R in the case of YU2^12^) sequences by replicating the design of pFUSE-hIgG1-Fc2, a publicly available plasmid sequence engineered for the construction of Fc-Fusion proteins (Invivogen, Cat # pfuse-hg1fc2); see also Supplementary Data 1 for Fc-fusion sequences. The Fc-fusion constructs were cloned into pVRC8400, expressed in 293F cells and then purified as we described for 17B. Here, affinity purification was by Protein A/G-agarose (Pierce, Cat #20398), followed by direct elution into 50 mM Tris, pH 8, using 1.5 column volumes of low pH IgG elution buffer (Pierce, Cat#21004), and then further separation and buffer exchange to PBS by size exclusion chromatography [Superdex 200 Increase 10/300 column (Cytiva, Cat# 289990944)]. Env-Fc versus Env-D368R-Fc signaling activities were first validated in VRC01 IgM BCR cells^12,78,86^ and then applied to monoclonal BCR cells expressing the gHgL and sHsL sequences identified in the present study.

BCR signaling was evaluated by calcium flux after incubating with multivalent antigen^78^. In these experiments, 1×10^6^ cells displaying monoclonal BCR were exposed to: 100 µg/ml Env-Fc; 100 µg/ml Env-D368R-Fc; or 1 μg/μl anti-IgM F(ab’)_2_ (Southern Biotech, Cat #2022-01). BCR stimulation was measured kinetically by flow cytometry (LSR II, BD) as the ratio of the Ca^2+^ bound/unbound states of the membrane permeable and ratiometric dye Fura Red. For each monoclonal BCR cell line, the ratiometric measurements were made before and after antigen exposure and the values were normalized to total Ca^2+^ flux capacity, as defined by exposure of the cells to 10 μg/ml ionomycin^78^.

### Production of monoclonal antibodies (mAbs) and Fabs

The variable region sequences from paired HC and LC sequences were cloned into human IgG1 HC and LC expression plasmids within pVRC8400^47,51^. The monoclonal antibodies were expressed and then purified using Protein A/G-agarose (Thermo Scientific, Cat #20398) and further separated by size exclusion chromatography [Superdex 200 Increase 10/300 column (Cytiva, Cat# 289990944)], as described for 17B. Fabs were generated by cleaving the purified IgGs using Endoproteinase Lys-C (New England Biolabs Cat. # P8109S)^51^. In this procedure, a mixture of 5 µg LysC per milligram of IgG1 was generated in PBS supplemented with 1 mM EDTA (Sigma, Cat # 03701). After 12 h at room temperature, a 1x complete protease inhibitor cocktail (Roche, Cat # 11697498001) was added to quench the reaction and uncleaved IgG1 was cleared by the addition of Protein A/G-agarose (Pierce, Cat #20398). The beads were washed in PBS and the supernatant was concentrated using Amicon Ultra concentrators (10 kDa MWCO, Millipore, Cat # UFC801008) and the Fabs were resolved on size exclusion FPLC using a Superdex 200 Increase 10/300 column (Cytiva, Cat# 289990944).

### Biolayer interferometry (BLI)

The binding affinities of gHgL, intermediate and sHsL forms of the Fabs were measured by biolayer interferometry using the Personal Assay BLItz System (Fortebio). Biotinylated avi-taged gp120s, generated as described earlier, were first loaded at 100 µg/mL onto streptavidin (SA) biosensors (Fortebio, Cat# 18-0009). After acquiring a 10 s baseline in 1xPBS, the Fabs (gHgL and sHsL) were applied at 1 µM, 2 µM, 5 µM, and 10 µM. The binding measurements used 60 s association and 30 s dissociation periods. The equilibrium dissociation constant (KD) values were then calculated by applying a 1:1 binding isotherm using the vendor-supplied software. KD values above 100 µM are beyond the limit of detection for this instrument^17^.

### Statistical analyses

All statistical analysis was performed using Prism Graphpad software. Sample sizes of animals and specific tests to determine statistical significance used are indicated in the figure legends. Probability values less than 0.05 were considered statistically significant.

### Reporting summary

Further information on research design is available in the Nature Portfolio Reporting Summary linked to this article.

### Supplementary information

Supplementary Information

Description of Additional Supplementary Files

Supplementary Data 1

Reporting Summary

### Source data

Source Data

## Data Availability

Source data are provided with this paper. Antibody sequences are deposited in GenBank and their accession numbers are listed in Supplementary Data 1. All other data are available in the article and its Supplementary files or from the corresponding author upon request Source data are provided with this paper.

## References

[1] Glanville J (2009). Precise determination of the diversity of a combinatorial antibody library gives insight into the human immunoglobulin repertoire. Proc. Natl. Acad. Sci. USA.

[2] Collins AM, Jackson KJL (2018). On being the right size: antibody repertoire formation in the mouse and human. Immunogenetics.

[3] Li Z, Woo CJ, Iglesias-Ussel MD, Ronai D, Scharff MD (2004). The generation of antibody diversity through somatic hypermutation and class switch recombination. Genes Dev..

[4] Victora GD, Nussenzweig MC (2012). Germinal centers. Annu. Rev. Immunol..

[5] Tas JM (2016). Visualizing antibody affinity maturation in germinal centers. Science.

[6] Mesin L, Ersching J, Victora GD (2016). Germinal center B cell dynamics. Immunity.

[7] Kuraoka M (2016). Complex antigens drive permissive clonal selection in germinal centers. Immunity.

[8] Radmacher MD, Kelsoe G, Kepler TB (1998). Predicted and inferred waiting times for key mutations in the germinal centre reaction: evidence for stochasticity in selection. Immunol. cell Biol..

[9] Silver J (2018). Stochasticity enables BCR-independent germinal center initiation and antibody affinity maturation. J. Exp. Med..

[10] Lingwood D (2012). Structural and genetic basis for development of broadly neutralizing influenza antibodies. Nature.

[11] McGuire AT (2014). HIV antibodies. Antigen modification regulates competition of broad and narrow neutralizing HIV antibodies. Science.

[12] Zhou T (2015). Structural repertoire of HIV-1-neutralizing antibodies targeting the CD4 supersite in 14 donors. Cell.

[13] Zhou T (2010). Structural basis for broad and potent neutralization of HIV-1 by antibody VRC01. Science.

[14] Bonsignori M (2018). Inference of the HIV-1 VRC01 antibody lineage unmutated common ancestor reveals alternative pathways to overcome a key glycan barrier. Immunity.

[15] Hoot S (2013). Recombinant HIV envelope proteins fail to engage germline versions of anti-CD4bs bNAbs. PLoS Pathog..

[16] McGuire AT, Glenn JA, Lippy A, Stamatatos L (2014). Diverse recombinant HIV-1 Envs fail to activate B cells expressing the germline B cell receptors of the broadly neutralizing anti-HIV-1 antibodies PG9 and 447-52D. J. Virol..

[17] Schmidt AG (2015). Immunogenic stimulus for germline precursors of antibodies that engage the influenza hemagglutinin receptor-binding site. Cell Rep..

[18] Skehel JJ, Wiley DC (2000). Receptor binding and membrane fusion in virus entry: the influenza hemagglutinin. Annu. Rev. Biochem..

[19] Hong M (2013). Antibody recognition of the pandemic H1N1 Influenza virus hemagglutinin receptor binding site. J. Virol..

[20] Zhou T (2013). Multidonor analysis reveals structural elements, genetic determinants, and maturation pathway for HIV-1 neutralization by VRC01-class antibodies. Immunity.

[21] Abbott RK (2018). Precursor frequency and affinity determine B cell competitive fitness in germinal centers, tested with germline-targeting HIV vaccine immunogens. Immunity.

[22] Sangesland M, Lingwood D (2021). Antibody focusing to conserved sites of vulnerability: The immunological pathways for ‘Universal’ influenza vaccines. Vaccines (Basel).

[23] Abbott RK, Crotty S (2020). Factors in B cell competition and immunodominance. Immunol. Rev..

[24] Stamatatos L, Pancera M, McGuire AT (2017). Germline-targeting immunogens. Immunol. Rev..

[25] Haynes BF (2023). Strategies for HIV-1 vaccines that induce broadly neutralizing antibodies. Nat. Rev. Immunol..

[26] Burton DR (2019). Advancing an HIV vaccine; advancing vaccinology. Nat. Rev. Immunol..

[27] Briney B (2016). Tailored immunogens direct affinity maturation toward HIV neutralizing antibodies. Cell.

[28] Tian M (2016). Induction of HIV neutralizing antibody lineages in mice with diverse precursor repertoires. Cell.

[29] McGuire AT (2016). Specifically modified Env immunogens activate B-cell precursors of broadly neutralizing HIV-1 antibodies in transgenic mice. Nat. Commun..

[30] Duan H (2018). Glycan masking focuses immune responses to the HIV-1 CD4-binding site and enhances elicitation of VRC01-class precursor antibodies. Immunity.

[31] Dosenovic P (2018). Anti-HIV-1 B cell responses are dependent on B cell precursor frequency and antigen-binding affinity. Proc. Natl. Acad. Sci. USA.

[32] Dosenovic P (2019). Anti-idiotypic antibodies elicit anti-HIV-1-specific B cell responses. J. Exp. Med..

[33] Jardine JG (2016). HIV-1 broadly neutralizing antibody precursor B cells revealed by germline-targeting immunogen. Science.

[34] Sok D (2016). Priming HIV-1 broadly neutralizing antibody precursors in human Ig loci transgenic mice. Science.

[35] Jardine JG (2015). Priming a broadly neutralizing antibody response to HIV-1 using a germline-targeting immunogen. Science.

[36] Dosenovic P (2015). Immunization for HIV-1 broadly neutralizing antibodies in human Ig knockin mice. Cell.

[37] Havenar-Daughton C (2018). The human naive B cell repertoire contains distinct subclasses for a germline-targeting HIV-1 vaccine immunogen. Sci. Transl. Med..

[38] Haynes BF, Burton DR, Mascola JR (2019). Multiple roles for HIV broadly neutralizing antibodies. Sci. Transl. Med..

[39] Medina-Ramirez M (2017). Design and crystal structure of a native-like HIV-1 envelope trimer that engages multiple broadly neutralizing antibody precursors in vivo. J. Exp. Med..

[40] Huang D (2020). B cells expressing authentic naive human VRC01-class BCRs can be recruited to germinal centers and affinity mature in multiple independent mouse models. Proc. Natl. Acad. Sci. USA.

[41] Chen X (2021). Vaccination induces maturation in a mouse model of diverse unmutated VRC01-class precursors to HIV-neutralizing antibodies with >50% breadth. Immunity.

[42] Scheid JF (2011). Sequence and structural convergence of broad and potent HIV antibodies that mimic CD4 binding. Science.

[43] Wu X (2011). Focused evolution of HIV-1 neutralizing antibodies revealed by structures and deep sequencing. Science.

[44] Leggat DJ (2022). Vaccination induces HIV broadly neutralizing antibody precursors in humans. Science.

[45] Caniels TG (2023). Germline-targeting HIV-1 Env vaccination induces VRC01-class antibodies with rare insertions. Cell Rep. Med..

[46] Steichen JM (2019). A generalized HIV vaccine design strategy for priming of broadly neutralizing antibody responses. Science.

[47] Sangesland M (2019). Germline-encoded affinity for cognate antigen enables vaccine amplification of a human broadly neutralizing response against Influenza virus. Immunity.

[48] Sangesland M (2020). A single human VH-gene allows for a broad-spectrum antibody response targeting bacterial Lipopolysaccharides in the blood. Cell Rep..

[49] Amitai A (2020). Defining and manipulating B cell immunodominance hierarchies to elicit broadly neutralizing antibody responses against Influenza virus. Cell Syst..

[50] Ronsard L (2021). Engineering an antibody V gene-selective vaccine. Front. Immunol..

[51] Sangesland M (2022). Allelic polymorphism controls autoreactivity and vaccine elicitation of human broadly neutralizing antibodies against influenza virus. Immunity.

[52] Caradonna TM (2022). An epitope-enriched immunogen expands responses to a conserved viral site. Cell Rep..

[53] Ingale J (2014). Hyperglycosylated stable core immunogens designed to present the CD4 binding site are preferentially recognized by broadly neutralizing antibodies. J. Virol..

[54] Wiehe K (2017). Immunodominance of antibody recognition of the HIV envelope V2 region in Ig-humanized mice. J. Immunol..

[55] Nishiyama Y, Planque S, Hanson CV, Massey RJ, Paul S (2012). CD4 binding determinant mimicry for HIV vaccine design. Front. Immunol..

[56] Wang S (2015). Manipulating the selection forces during affinity maturation to generate cross-reactive HIV antibodies. Cell.

[57] Shaffer JS, Moore PL, Kardar M, Chakraborty AK (2016). Optimal immunization cocktails can promote induction of broadly neutralizing Abs against highly mutable pathogens. Proc. Natl. Acad. Sci. USA.

[58] Kwon YD (2012). Unliganded HIV-1 gp120 core structures assume the CD4-bound conformation with regulation by quaternary interactions and variable loops. Proc. Natl. Acad. Sci. USA.

[59] Thali M (1991). Characterization of a discontinuous human immunodeficiency virus type 1 gp120 epitope recognized by a broadly reactive neutralizing human monoclonal antibody. J. Virol..

[60] Lynch RM (2012). The development of CD4 binding site antibodies during HIV-1 infection. J. Virol..

[61] Li Y (2007). Broad HIV-1 neutralization mediated by CD4-binding site antibodies. Nat. Med..

[62] Li Y (2009). Analysis of neutralization specificities in polyclonal sera derived from human immunodeficiency virus type 1-infected individuals. J. Virol..

[63] Torrents de la Pena A (2018). Immunogenicity in rabbits of HIV-1 SOSIP trimers from clades A, B, and C, given individually, sequentially, or in combination. J. Virol..

[64] Klasse PJ (2016). Sequential and simultaneous immunization of rabbits with HIV-1 envelope glycoprotein SOSIP.664 trimers from clades A, B and C. PLoS Pathog..

[65] Tan HX (2019). Subdominance and poor intrinsic immunogenicity limit humoral immunity targeting influenza HA stem. J. Clin. Investig..

[66] Lofano G (2018). Antigen-specific antibody Fc glycosylation enhances humoral immunity via the recruitment of complement. Sci. Immunol..

[67] Olsson J (2019). Urea dilution of serum for reproducible anti-HSV1 IgG avidity index. BMC Infect. Dis..

[68] Reinhardt RL, Liang HE, Locksley RM (2009). Cytokine-secreting follicular T cells shape the antibody repertoire. Nat. Immunol..

[69] Victora GD (2010). Germinal center dynamics revealed by multiphoton microscopy with a photoactivatable fluorescent reporter. Cell.

[70] Kepler TB (2013). Reconstructing a B-cell clonal lineage. I. Statistical inference of unobserved ancestors. F1000Res.

[71] Kepler TB (2014). Reconstructing a B-cell clonal lineage. II. Mutation, selection, and affinity maturation. Front. Immunol..

[72] McCarthy KR (2018). Memory B cells that cross-react with group 1 and group 2 Influenza A viruses are abundant in adult human repertoires. Immunity.

[73] Ferdman J (2018). Intra-seasonal antibody repertoire analysis of a subject immunized with an MF59(R)-adjuvanted pandemic 2009 H1N1 vaccine. Vaccine.

[74] Bajic G (2019). Influenza antigen engineering focuses immune responses to a subdominant but broadly protective viral epitope. Cell Host microbe.

[75] Williams WB (2021). Fab-dimerized glycan-reactive antibodies are a structural category of natural antibodies. Cell.

[76] Ramesh A (2017). Structure and diversity of the rhesus Macaque immunoglobulin loci through multiple de novo genome assemblies. Front. Immunol..

[77] Jenni S (2022). Rotavirus VP4 epitope of a broadly neutralizing human antibody defined by its structure bound with an attenuated-strain virion. J. Virol..

[78] Weaver GC (2016). In vitro reconstitution of B cell receptor-antigen interactions to evaluate potential vaccine candidates. Nat. Protoc..

[79] Yassine HM (2015). Hemagglutinin-stem nanoparticles generate heterosubtypic influenza protection. Nat. Med..

[80] Villar RF (2016). Reconstituted B cell receptor signaling reveals carbohydrate-dependent mode of activation. Sci. Rep..

[81] Veneziano R (2020). Role of nanoscale antigen organization on B-cell activation probed using DNA origami. Nat. Nanotechnol..

[82] Tokatlian T (2018). Enhancing humoral responses against HIV envelope trimers via nanoparticle delivery with stabilized synthetic liposomes. Sci. Rep..

[83] Tokatlian T (2019). Innate immune recognition of glycans targets HIV nanoparticle immunogens to germinal centers. Science.

[84] Saunders KO (2019). Targeted selection of HIV-specific antibody mutations by engineering B cell maturation. Science.

[85] Corbett KS (2019). Design of nanoparticulate group 2 Influenza virus hemagglutinin stem antigens that activate unmutated ancestor B cell receptors of broadly neutralizing antibody lineages. mBio.

[86] Setliff I (2019). High-throughput mapping of B cell receptor sequences to antigen specificity. Cell.

[87] Feldman J (2021). Naive human B cells engage the receptor binding domain of SARS-CoV-2, variants of concern, and related sarbecoviruses. Sci. Immunol..

[88] Shlomchik MJ (2018). Do memory B cells form secondary germinal centers? yes and no. Cold Spring Harb. Perspect. Biol..

[89] Mesin L (2020). Restricted clonality and limited germinal center reentry characterize memory B cell reactivation by boosting. Cell.

[90] Sabouri Z (2014). Redemption of autoantibodies on anergic B cells by variable-region glycosylation and mutation away from self-reactivity. Proc. Natl. Acad. Sci. USA.

[91] Hagglof T (2023). Continuous germinal center invasion contributes to the diversity of the immune response. Cell.

[92] de Carvalho RVH (2023). Clonal replacement sustains long-lived germinal centers primed by respiratory viruses. Cell.

[93] Yu L, Guan Y (2014). Immunologic basis for long HCDR3s in broadly neutralizing antibodies against HIV-1. Front. Immunol..

[94] Rettig TA, Ward C, Bye BA, Pecaut MJ, Chapes SK (2018). Characterization of the naive murine antibody repertoire using unamplified high-throughput sequencing. PloS One.

[95] Saunders KO (2022). Stabilized HIV-1 envelope immunization induces neutralizing antibodies to the CD4bs and protects macaques against mucosal infection. Sci. Transl. Med..

[96] Stephenson KE, Wagh K, Korber B, Barouch DH (2020). Vaccines and broadly neutralizing antibodies for HIV-1 prevention. Annu. Rev. Immunol..

[97] Finney J, Kelsoe G (2018). Poly- and autoreactivity of HIV-1 bNAbs: implications for vaccine design. Retrovirology.

[98] Guthmiller JJ (2020). Polyreactive broadly neutralizing B cells are selected to provide defense against pandemic threat Influenza viruses. Immunity.

[99] Tam HH (2016). Sustained antigen availability during germinal center initiation enhances antibody responses to vaccination. Proc. Natl. Acad. Sci. USA.

[100] Lee JH (2022). Long-primed germinal centres with enduring affinity maturation and clonal migration. Nature.

[101] Kanekiyo M (2013). Self-assembling influenza nanoparticle vaccines elicit broadly neutralizing H1N1 antibodies. Nature.

[102] de Souza VA (2004). Use of an immunoglobulin G avidity test to discriminate between primary and secondary dengue virus infections. J. Clin. Microbiol..

[103] Trombetta, J. J. et al. Preparation of Single-Cell RNA-Seq Libraries for Next Generation Sequencing. *Current protocols in molecular biology / edited by F*rederick *M*. *Ausubel… [et al.]* **107**, 4 22 21-24 22 17, 10.1002/0471142727.mb0422s107 (2014). 10.1002/0471142727.mb0422s107 PMC4338574 24984854

[104] Masella AP, Bartram AK, Truszkowski JM, Brown DG, Neufeld JD (2012). PANDAseq: paired-end assembler for illumina sequences. BMC Bioinforma..

[105] Shi B (2014). Comparative analysis of human and mouse immunoglobulin variable heavy regions from IMGT/LIGM-DB with IMGT/HighV-QUEST. Theor. Biol. Med. Model..

[106] Tamura K (2011). MEGA5: molecular evolutionary genetics analysis using maximum likelihood, evolutionary distance, and maximum parsimony methods. Mol. Biol. Evolut..

[107] Tamura K, Stecher G, Kumar S (2021). MEGA11: Molecular evolutionary genetics analysis Version 11. Mol. Biol. Evolut..

