# Supplementary material for: Engaging an HIV vaccine target through the acquisition of low B cell affinity
Source: Nat Commun. 2023 Aug 28;14:5249. doi: 10.1038/s41467-023-40918-2 (PMC10462694; doi:10.1038/s41467-023-40918-2)
Supplement: Supplementary file 1 — Supplementary Information [file 41467_2023_40918_MOESM1_ESM.pdf]

**Supplementary Information**

# Engaging an HIV vaccine target through the acquisition of low B cell affinity

Larance Ronsard[1]*, Ashraf S. Yousif[1]*, Faez Amokrane Nait Mohamed[1], Jared Feldman[1], Vintus Okonkwo[1], Caitlin McCarthy[1], Julia Schnabel[1],Timothy Caradonna[1], Ralston M. Barnes[2], Daniel Rohrer[2], Nils Lonberg[2], Aaron Schmidt[1,3] and Daniel Lingwood[1]

[1]The Ragon Institute of Mass General, The Massachusetts Institute of Technology and Harvard University, 400 Technology Square, Cambridge, MA 02139

[2]Bristol-Myers Squibb, 700 Bay Rd, Redwood City, California, 94063-2478

[3]Department of Microbiology, Harvard Medical School, Boston, MA 02115, USA

*equal contribution

**Correspondence:**

Daniel Lingwood

Tel: 857-268-7180; E-mail: dlingwood@mgh.harvard.edu

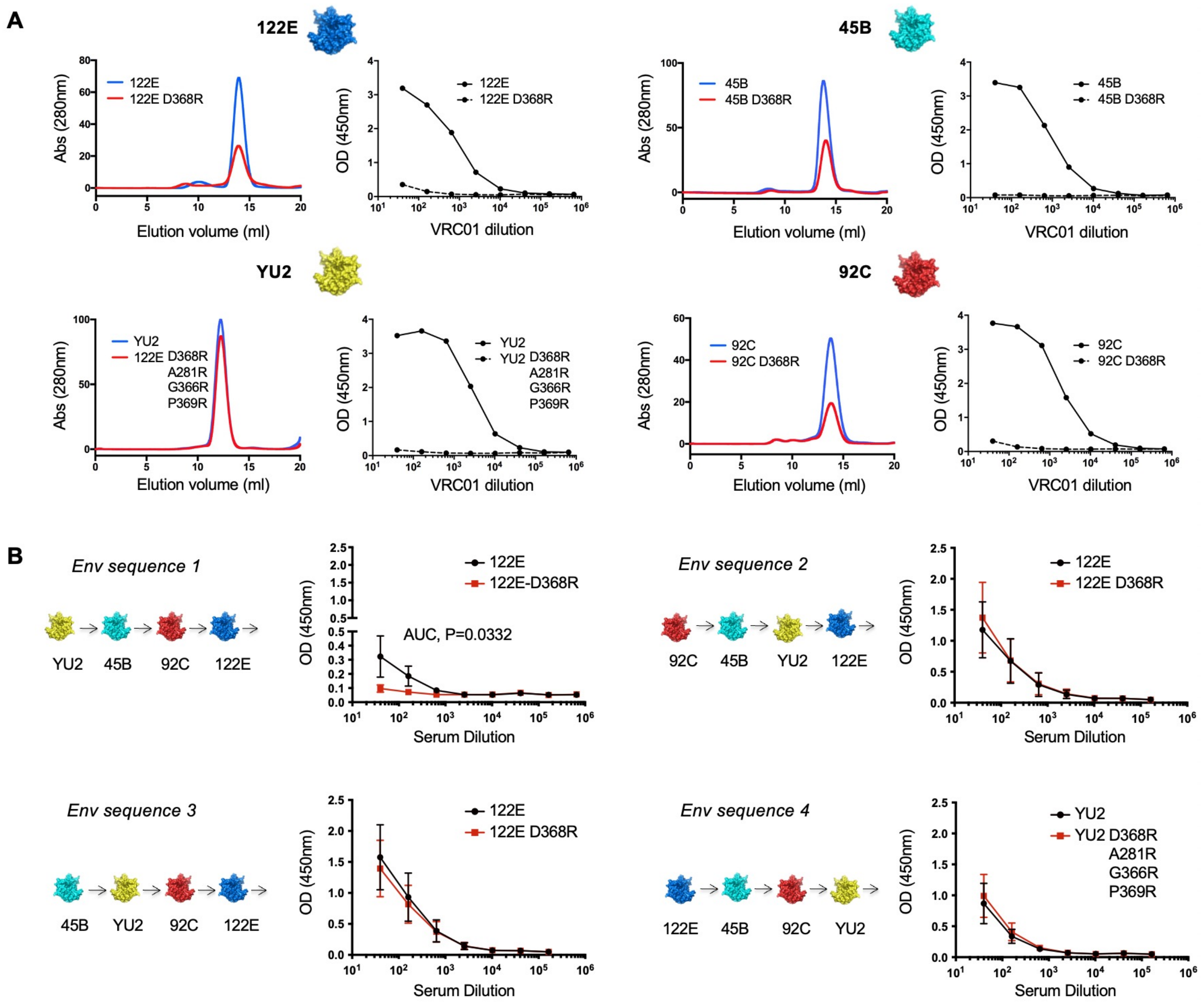


**Supplementary Figure 1**. **Recombinant Env monomers and screening for immunization order in WT C57Bl/6 mice.** (A) Affinity tag-free Env monomers were purified on 17B columns and quality controlled using by size exclusion chromatography (superdex increase 10/300). D368-dependent antigenicity to VRC01 was confirmed in ELISA. Tagless Envs were deployed as the vaccine immunogens. WT and D368R (D368R, A281R,G366R, P369R in the case of YU2) Envs were also made with the Avi Tag, a site specific biotinylatlon target, and were deployed as B cell flow cytometry probes following fluorophore conjugation. (B) Env immunization sequences 1-4 were applied in wildtype C57Bl/6 and the reactivity of the serum antibodies targeting the terminal Env sequence (122E vs 122E-D368R for immunization sequences 1-3 and YU2 vs YU2-(D368R, A281R,G366R, P369R) for immunization sequence 4) was recorded after the final immunization step (mean ± SD, n=5 biologically independent animals per immunization sequence; the WT vs D368R curves were compared by area under the curve, P=0.0332, two-sided paired T test of ratio). Immunization sequence 1 was chosen to deploy in IGHV1-2 HC2 mice.

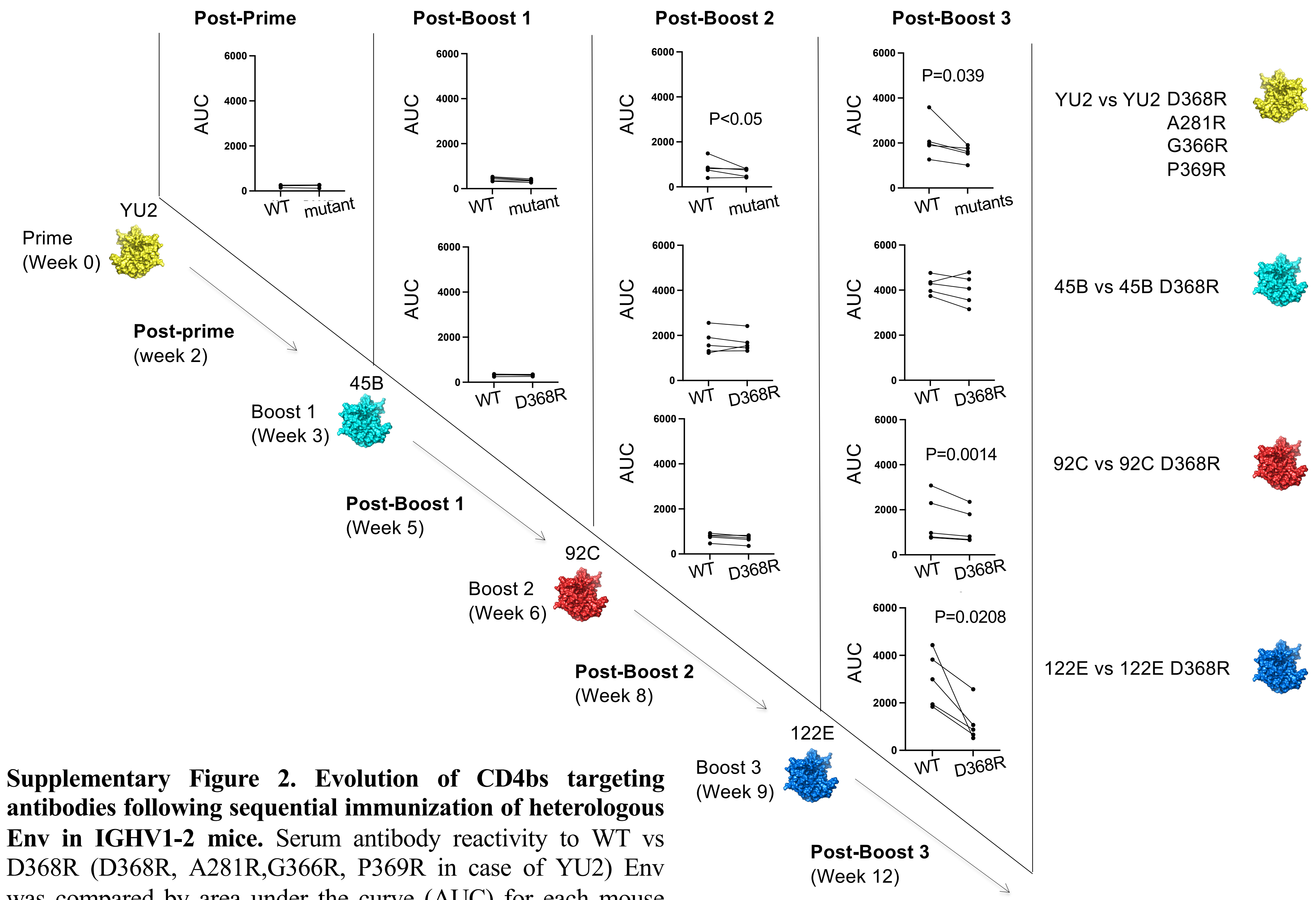


**Supplementary Figure 2. Evolution of CD4bs targeting antibodies following sequential immunization of heterologous Env in IGHV1-2 mice.** Serum antibody reactivity to WT vs D368R (D368R, A281R,G366R, P369R in case of YU2) Env was compared by area under the curve (AUC) for each mouse two weeks after inoculation with each Env strain (n=5 biologically independent animals, P values are indicated in the image, two-sided paired T test of ratio).

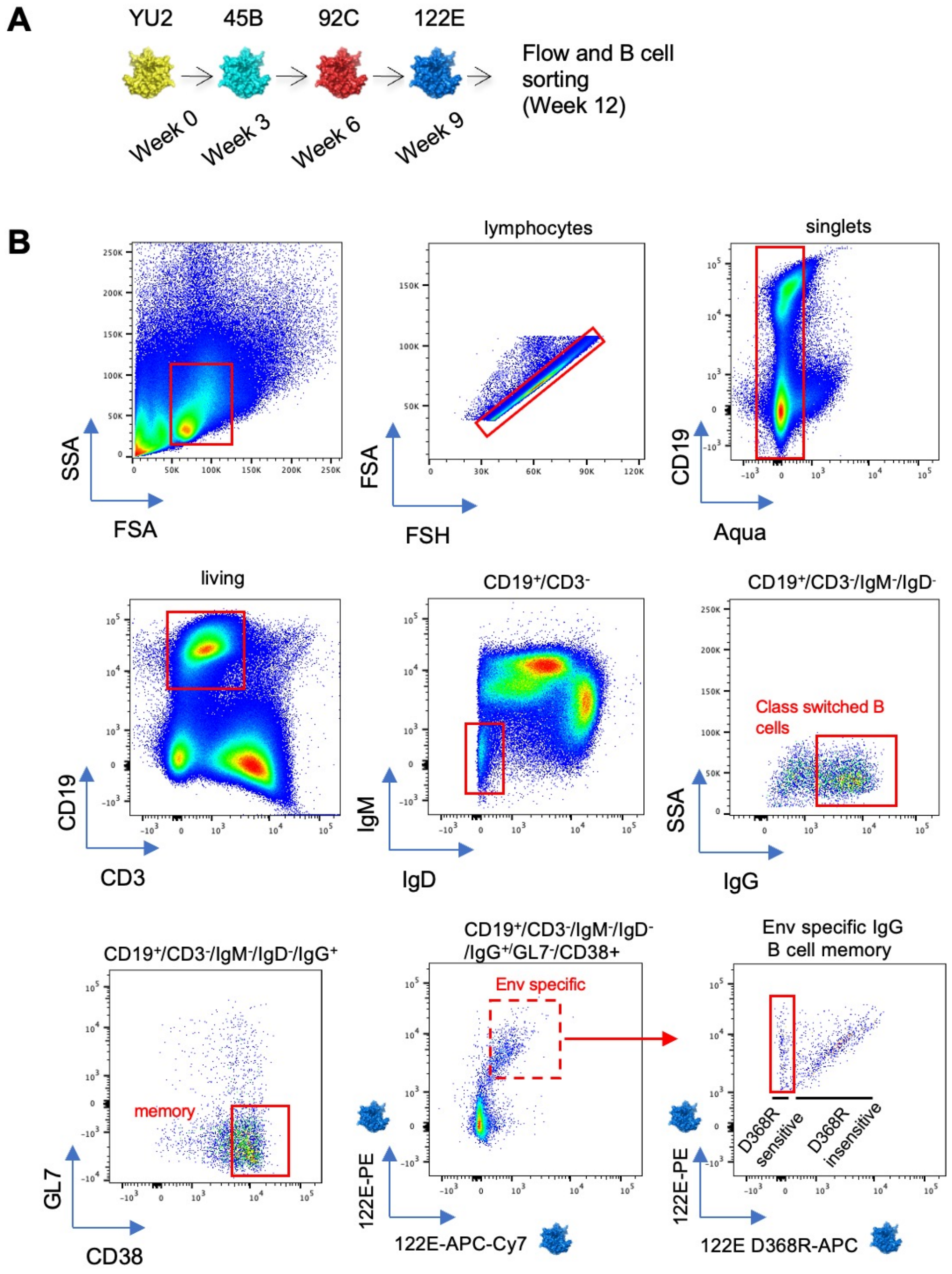


**Supplementary Figure 3. Gating for IgG memory B cells from spleen following sequential immunization Env**. (A) Heterologous immunization regimen and flow cytometry and sorting time point. (B) Gating strategy for flow cytometry and single cell FACS of Env-specific IgG memory B cells with D368R-sensitivity ($CD3^-/CD19^+/IgM^-/IgD^-/IgG^+/GL7^-/CD38^+$/Env-$PE^+$/Env-APC-$Cy7^+$/Env-D368R-$APC^-$).

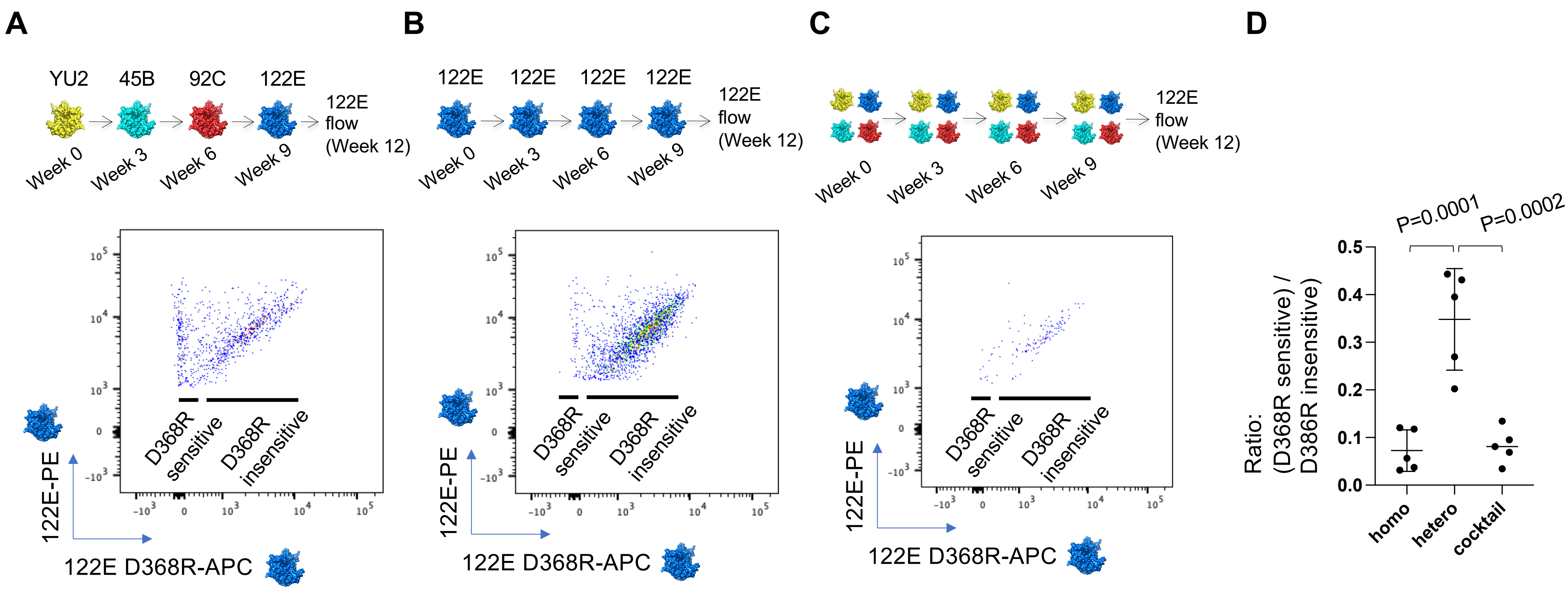


**Supplementary Figure 4. Sequential immunization with the antigen cocktail generates elicits lower D368R-sensitive memory B cells.** (A, B) Env-specific IgG memory B cells from spleen (gated on: $CD3^-/CD19^+/IgM^-/IgD^-/IgG^+/GL7^-/CD38^+$/122E Env-$PE^+$/ 122E Env-APC-$Cy7^+$) expanded following sequential immunization with homologous (homo) or heterologous (hetero) Env (data from Figure 1E,F). (C) Env-specific IgG memory B cells following sequential immunization of all for Env antigens as a cocktail (gated on: $CD3^-/CD19^+/IgM^-/IgD^-/IgG^+/GL7^-/CD38^+$/122E Env-$PE^+$/ 122E Env-APC-$Cy7^+$). (D) Ratio of D368R sensitive to D368R insensitive IgG B cells expanded by the vaccination regimens [mean ± SD, n=5 biologically independent animals, P=0.0001 (homologous vs heterologous), P=0.0002 (heterologous vs cocktail), ANOVA with Tukey's test]

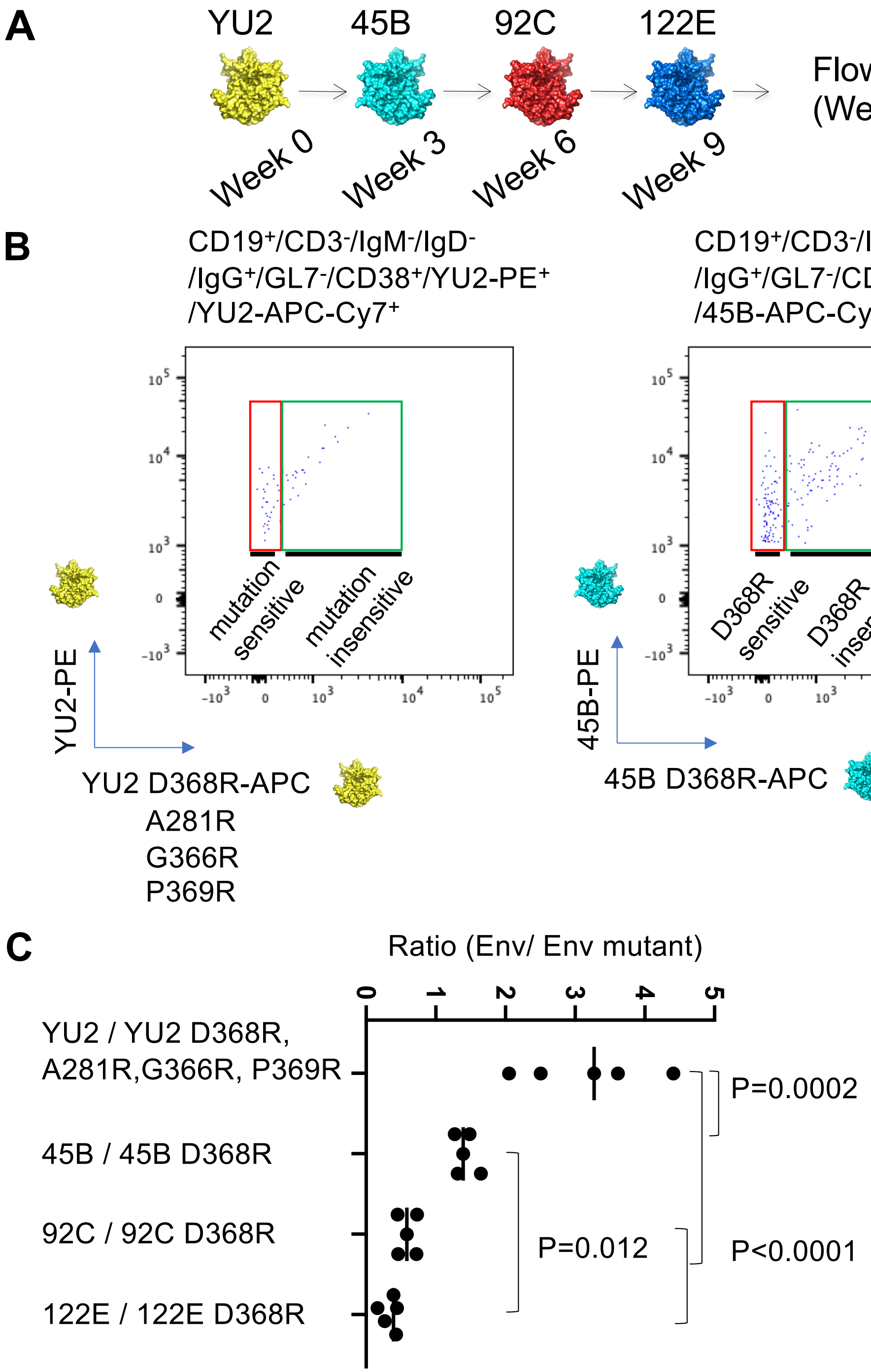


**Supplementary Figure 5. After heterologous immunization, the IgG memory B cells expanded in the spleen show higher D368R insensitivity for recently immunized Env strains, and higher D368R sensitivity for less recently immunized Env strains. (A)** The heterologous immunization regimen. (B) After the final immunization, Env-specific IgG memory B cells ($CD3^-/CD19^+/IgM^-/IgD^-/IgG^+/GL7^-/CD38^+/Env\text{-}PE^+/Env\text{-}APC\text{-}Cy7^+$; see Supplementary Figure 3 for gating) were identified as mutation-sensitive versus mutation-insensitive using flow probes for each Env strain (YU2-PE, YU2-APC-Cy7, YU2-(D368R, A281R, G366R, P369R)-APC; 45B-PE, 45B-APC-Cy7, 45B-D368R-APC; 92C-PE, 92C-APC-Cy7, 92C-D368R-APC; 122E-PE, 122E-APC-Cy7, 122E-D368R-APC). (C) Ratio of D368R-sensitive versus D368R-insensitive as measured for each Env strain (mean, n=5 biologically independent animals, P values indicated on image, ANOVA with Tukey's test).

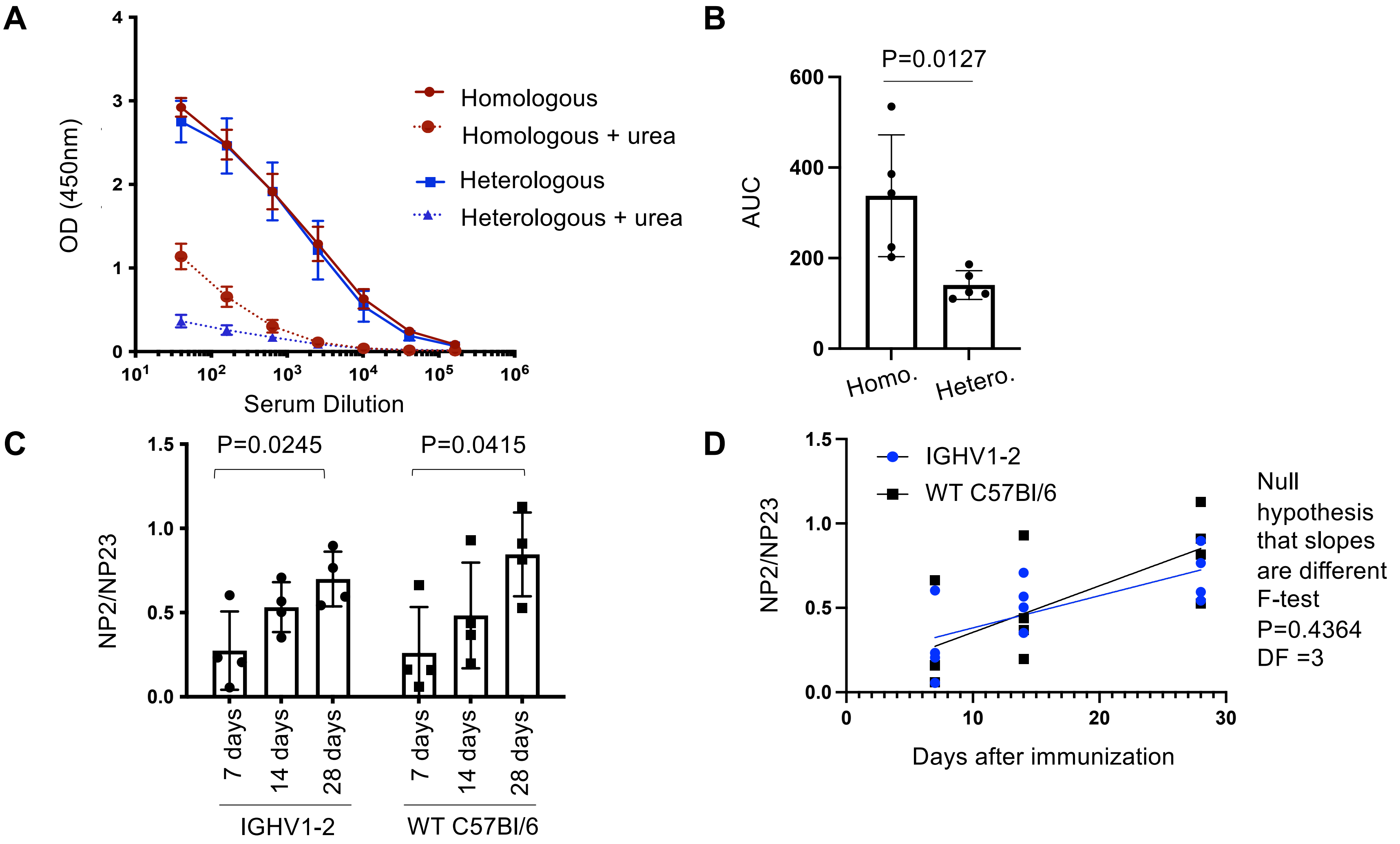


**Supplementary Figure 6. Lowered binding strength accompanies D368R sensitivity within the serum antibody response**. (A) Same immune sera as in Figure 1B and Figure 1D except binding to 122E Env occurred in presence or absence of urea (mean ± SD, n=5 biologically independent animals per immunization regimen). (B) Area under the curve (AUC) comparison for homologous and heterologous regimen in the presence of urea (AUC, n=5 biologically independent animals per immunization regimen, P=0.0127, Two-sided Student's T-test). (C) To confirm that affinity maturation was operable in the transgenic animals, both IGHV1-2 and WT C57Bl/6 mice were immunized with NP-ovalbumin and the accumulating increases in the NP2/N23 binding due to affinity maturation were recorded in the serum antibody response (mean ± SD, n=4 biologically independent animals per genotype, P=0.0245 (7 days vs 28 days, IGHV1-2 mice), P=0.0415 (7 days vs 28 days, WT C57Bl/6 mice), ANOVA with Tukey's test). (D) Same data as in (C) regressed over the post-immunization time period. The rates of increase in the NP2/N23 ratios were indistinguishable between the IGHV1-2 and WT C57Bl/6 mice (n=4 biologically independent animals per genotype, P=0.4364, linear regression with F-test for comparison of slopes).

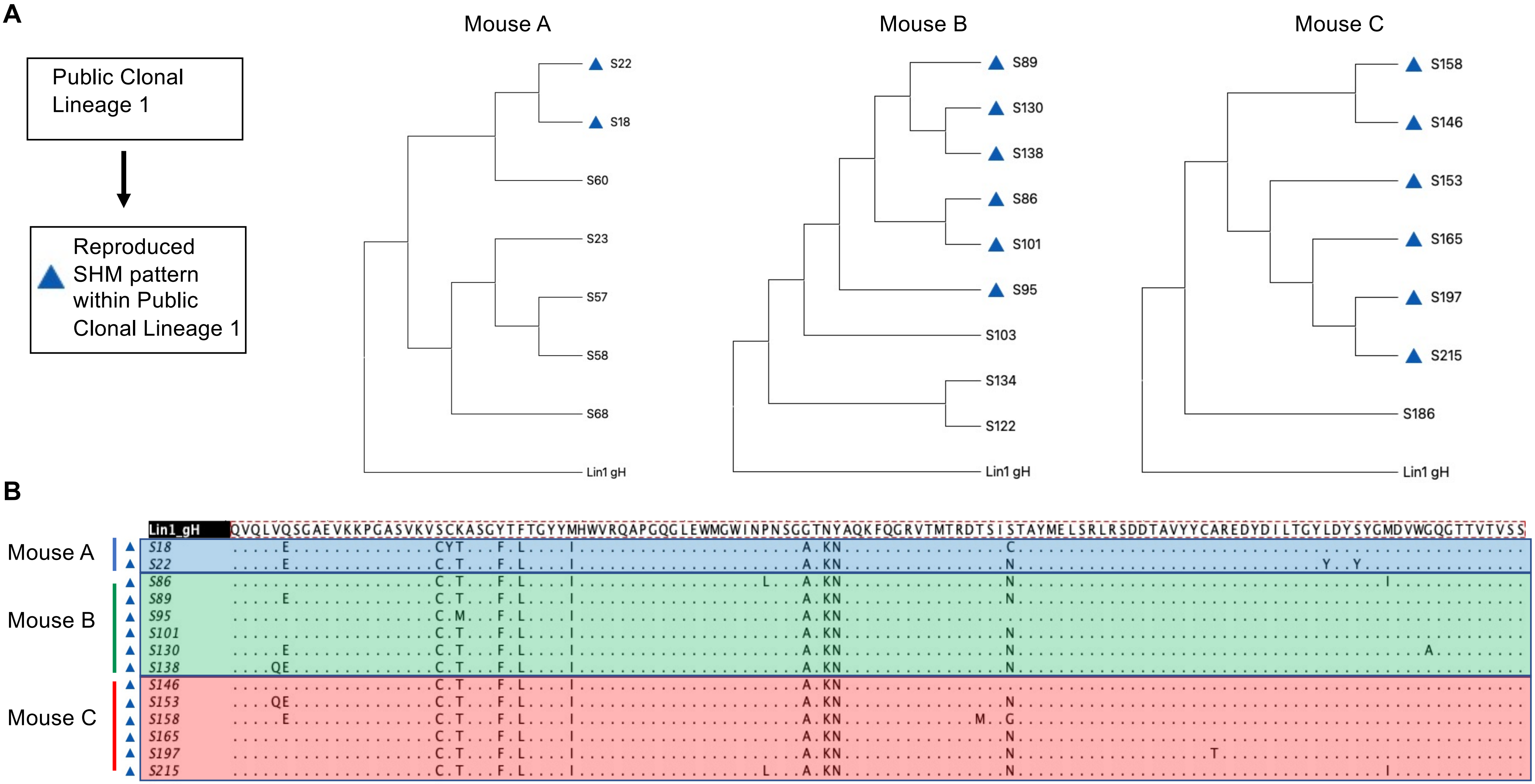


Representative sHsL for Lin1 (bearing shared core pattern in HC SHM) = S89

**Supplementary Figure 7. Reproduced Patterns of Somatic Hypermutation within Public Clonal Lineage 1.** Public clonal lineages are defined as public CDRH3 + shared CDRL3 (see also Figure 2 and Supplementary Data 1). (A) Phylogeny of Public clonal lineage 1 by HC nucleotide sequence. Clones marked by a blue triangle identify BCR sequences with convergent SHM pattern within the HC, which was independently reproduced across the different mice. (B) Corresponding amino acid sequences of the clonal B cells. S89 was chosen as a representative sHsL BCR sequence for Lin1 because it contains a core SHM pattern shared across the mice. The phylogeny of inferred intermediates within Lin1 is depicted later in Supplementary Figure 12 and in the context of all the BCR sequences within Public Clonal Lineage 1.

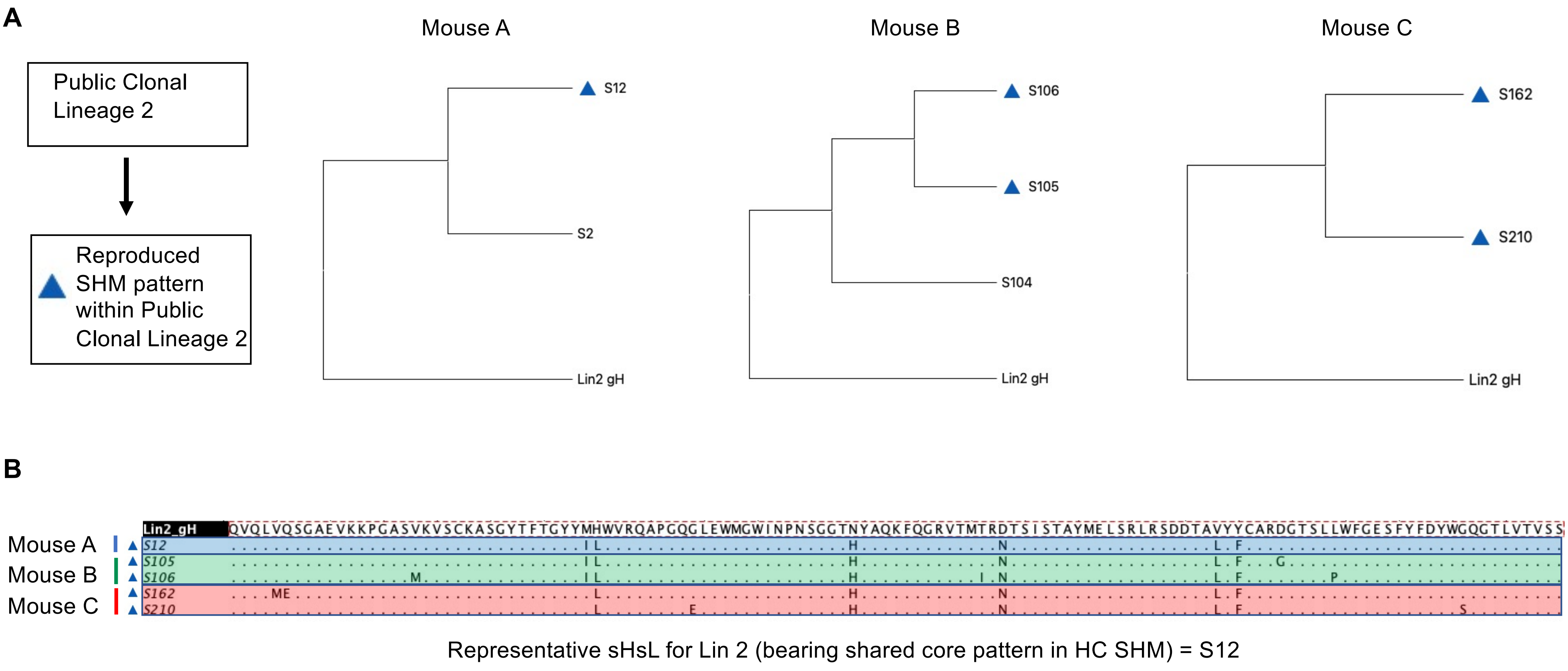


**Supplementary Figure 8. Reproduced Patterns of Somatic Hypermutation within Public Clonal Lineage 2.** Public clonal lineages are defined as public CDRH3 + shared CDRL3 (see also Figure 2 and Supplementary Data 1). (A) Phylogeny of Public clonal lineage 2 by HC nucleotide sequence. Clonal B cells marked by a blue triangle identify BCR sequences with convergent SHM pattern within the HC, which were independently reproduced across the different mice. (B) Corresponding amino acid sequences of the clonal B cells. S12 was chosen as a representative sHsL BCR sequence for Lin2 because it contains a core SHM pattern shared across the mice. The phylogeny of inferred intermediates within Lin2 is depicted later in Supplementary Figure 16 and in the context of all the BCR sequences within Public Clonal Lineage 2.

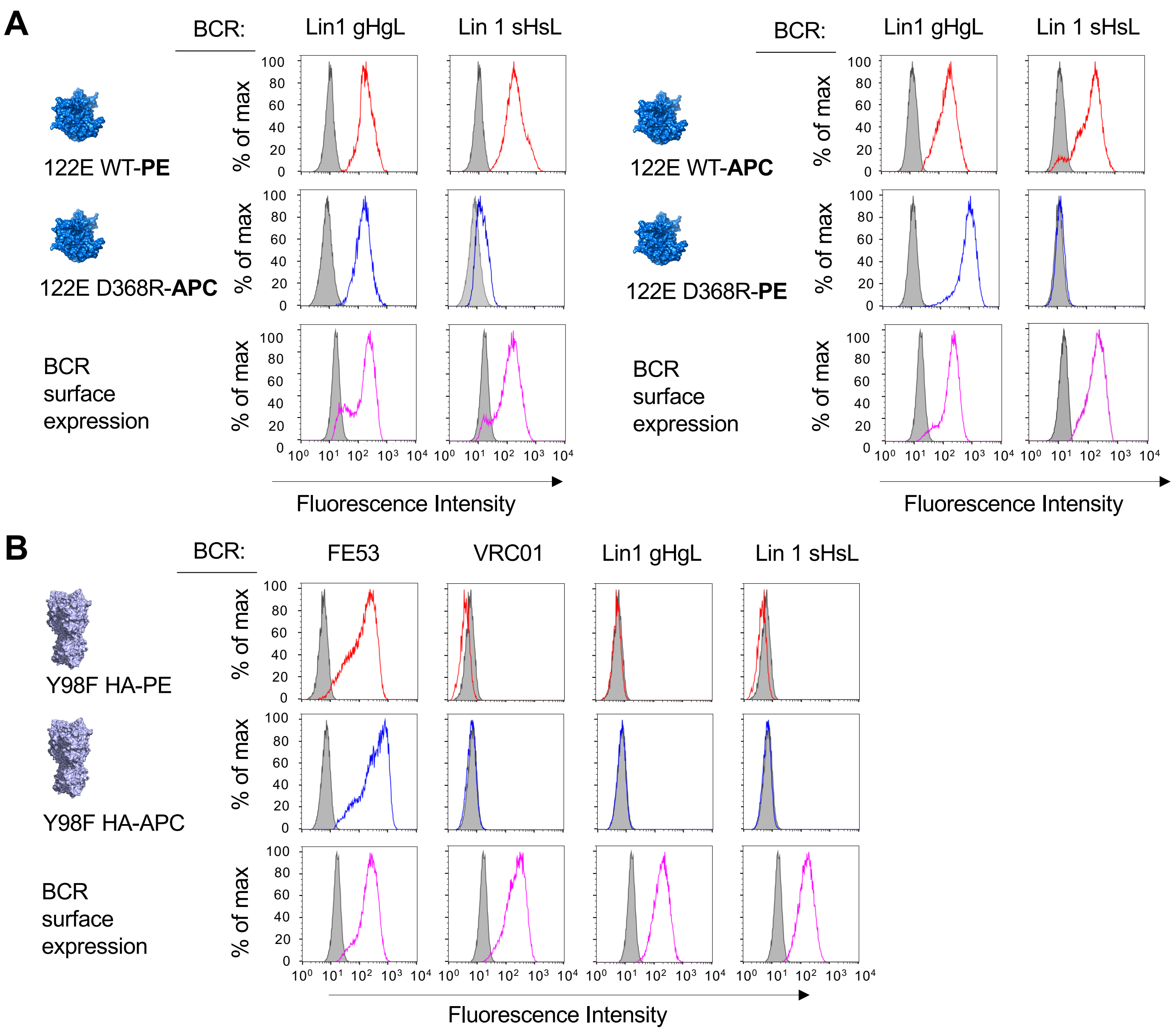


**Supplementary Figure 9. Lin1 BCR antigenicity is the same after reversing the antigen colors.** (A) 122E Env recognition by BCRs: VRC01; Lin1 gHgL; Lin1 sHsL. Binding with fluorescently labelled 122E (Left panel = WT-PE vs D368R-APC vs right panel = WT-APC vs D368R-PE ) was measured by flow cytometry for the BCR of interest (red = WT; blue = D368R). Grey is binding to an isotype control (for 122E binding) or binding to surface BCR negative (for surface BCR expression). BCR surface expression was measured by anti-light chain reactivity (pink). (B) BCR binding to irrelevant Y98F influenza hemagglutinin (HA) probe that has been labeled with either PE or APC. Binding is to FE53 BCR, an HA-targeting BCR or to VRC01, Lin1 gHgL, or Lin1 sHsL (grey = binding to BCR-surface negative).

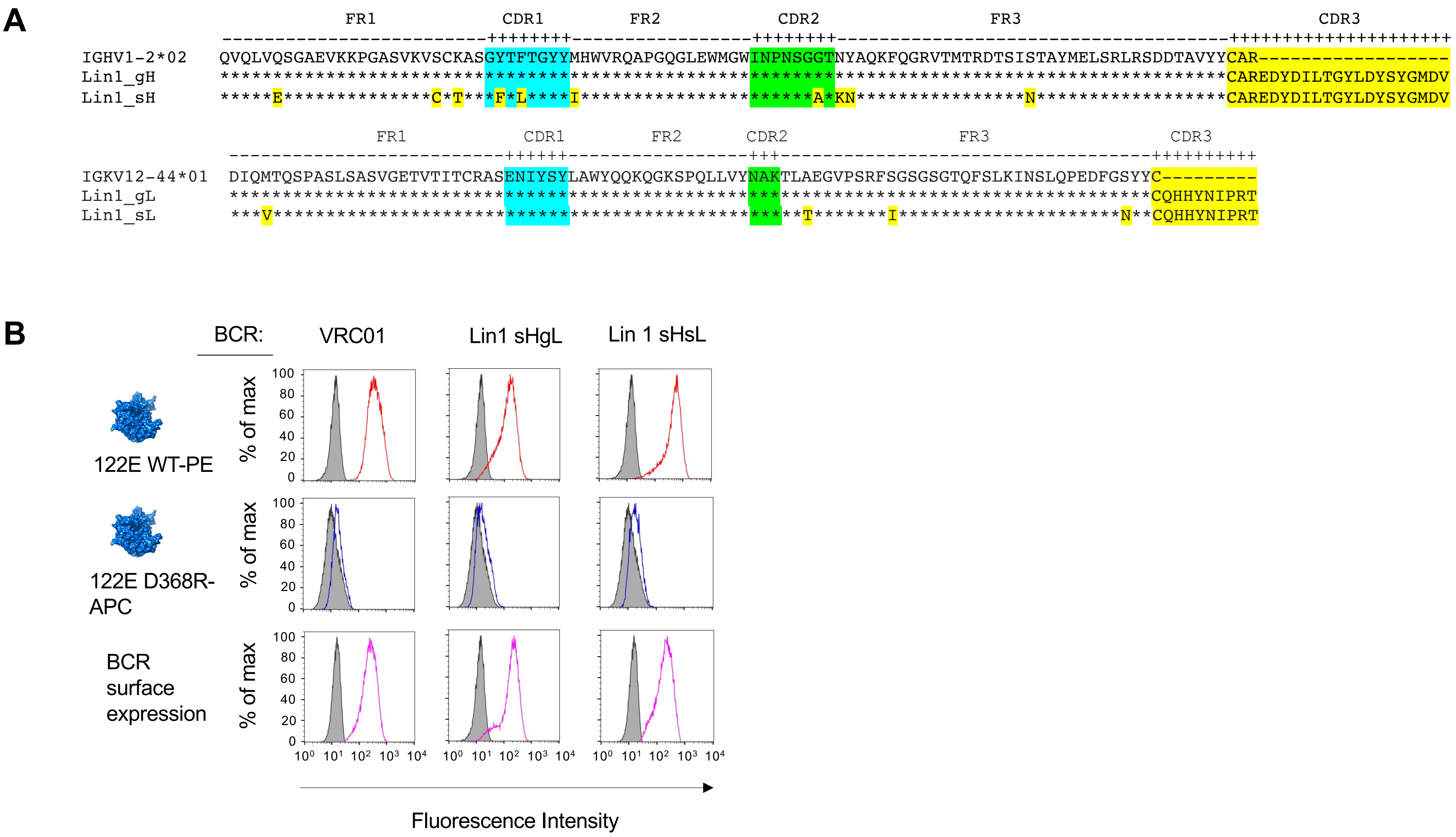


**Supplementary Figure 10. HC directed acquisition of D368R sensitivity by Lin1.** (A) gH, sH, gL, sL sequences of the public clone Lin1. (B) 122E Env recognition by BCRs: VRC01; Lin1 sHgL; Lin1 sHsL. Binding with fluorescently labelled 122E (WT-PE vs D368R-APC) was measured by flow cytometry for the BCR of interest [red = WT; blue = D368R) and grey for isotype control (for 122E binding) or grey for surface BCR negative (for surface BCR expression)]. BCR surface expression was measured by anti-light chain reactivity (pink).

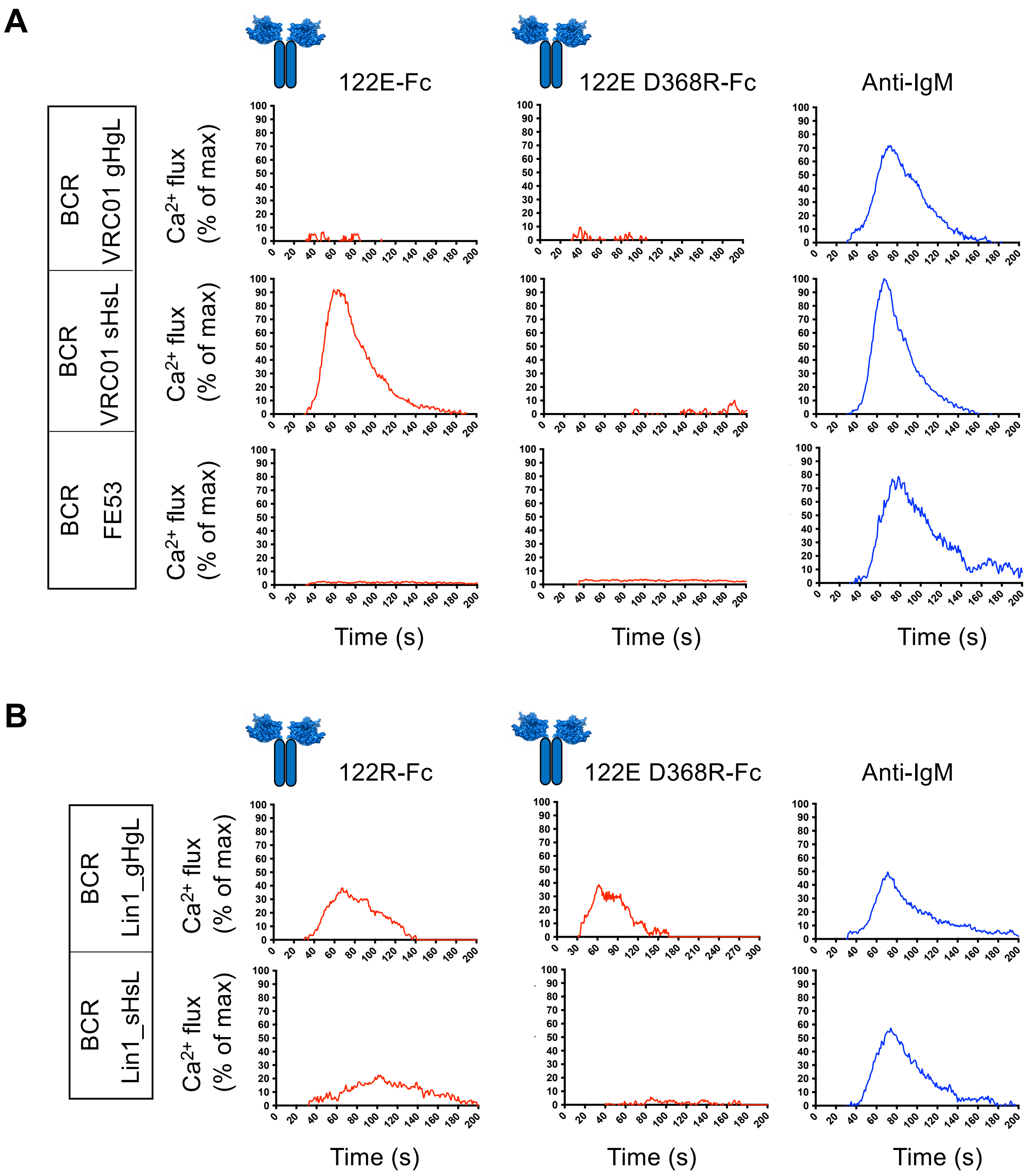


**Supplementary Figure 11. Isotype controls and Lin1 BCR triggering through 122E-Fc vs 122E D368R-Fc. (A)** $Ca^{2+}$ flux activity, measured kinetically on three B cell lines presenting IgM BCRs of known antigenicity: VRC01 gHgL (non-envelope specific); VRC01 (Env specific and D368R sensitive); and FE53 (specific for hemagglutinin from influenza virus). **(B)** Lin1 gHgL BCR vs Lin1 sHsL BCR triggering in response to bivalent 122E-Fc and/or 122E -D368R-Fc or anti-IgM. In all cases Fluxing was measured by the ratiometric $Ca^{2+}$ sensing dye fura red and normalized to total flux capacity, as defined by the ionophore ionomycin.

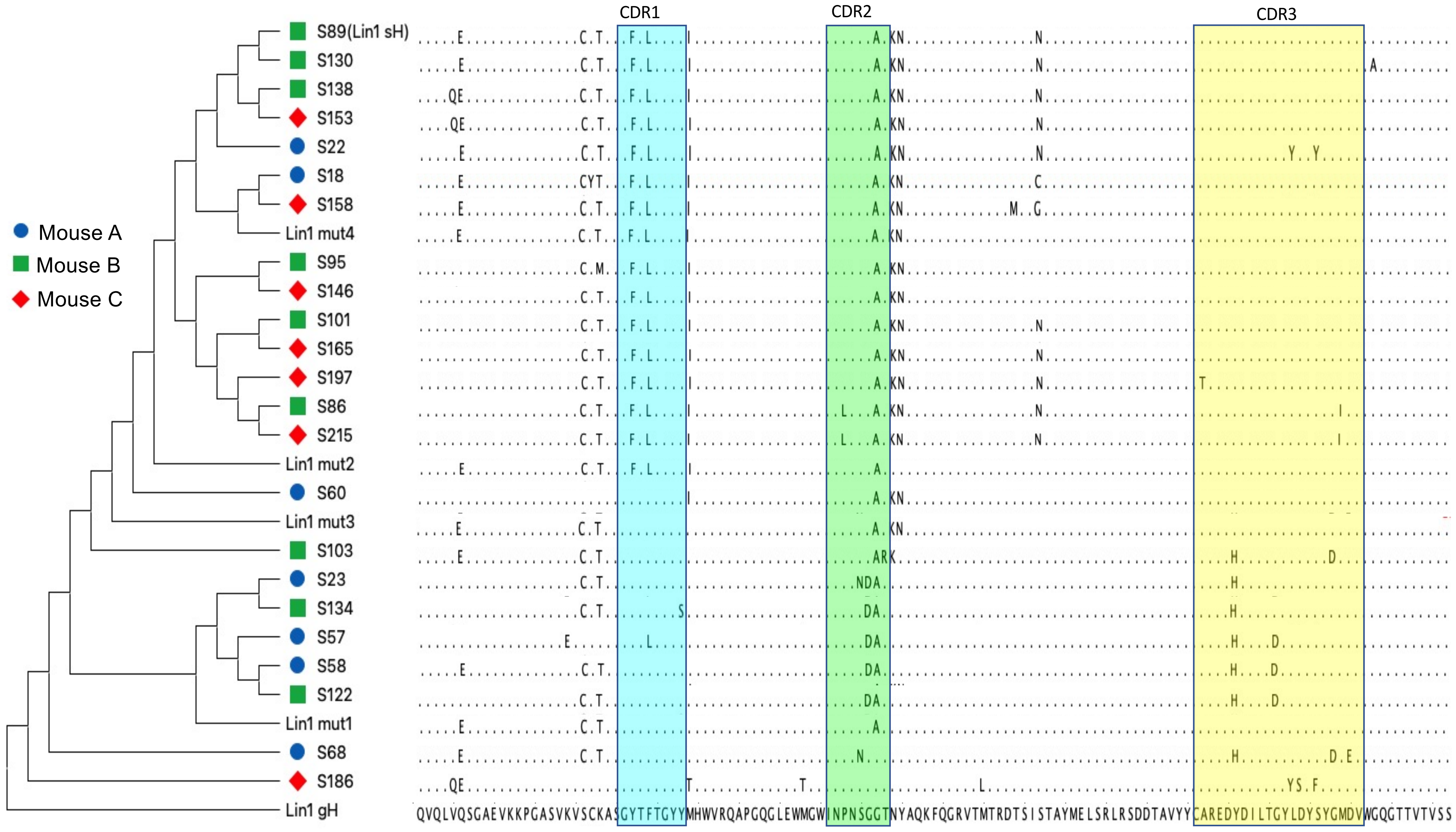


**Supplementary Figure 12. Phylogenic of Public Clonal Lineage 1 with inferred intermediates.** Public clonal lineages are defined as public CDRH3 + shared CDRL3 (see also Figure 2B and Supplementary Data 1)**.** Phylogeny of Public Clonal Lineage 1 by HC nucleotide sequence. To broadly highlight where the inferred intermediates (Lin1_mut1-4) distribute within the public pathway, the tree was constructed using all the Public Lineage 1 clones from the three vaccine recipients.

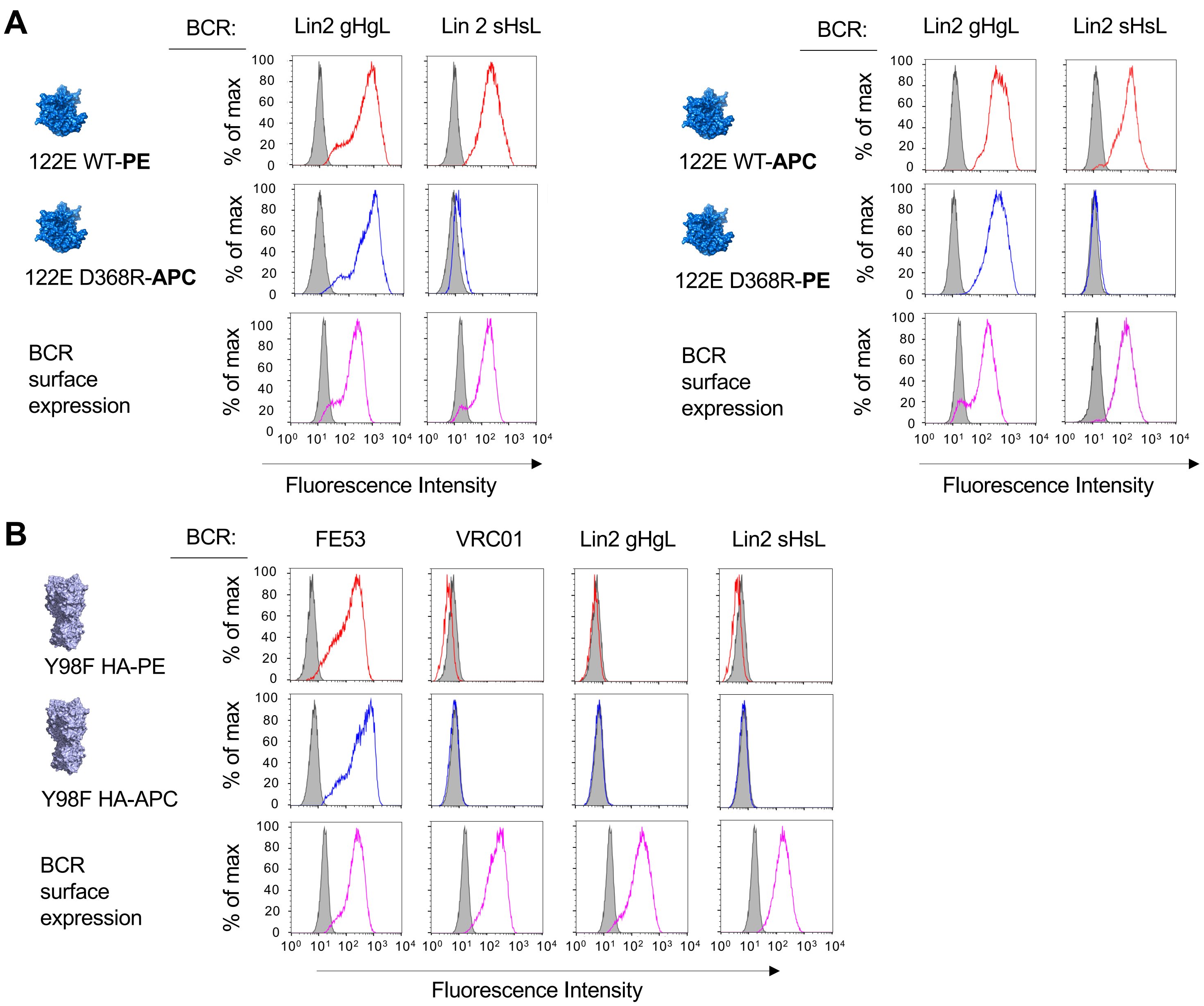


**Supplementary Figure 13. Lin2 BCR antigenicity is the same after reversing the antigen colors** (A) 122E Env recognition by BCRs: VRC01; Lin2 gHgL; Lin2 sHsL. Binding with fluorescently labelled 122E (Left panel = WT-PE vs D368R-APC vs right panel = WT-APC vs D368R-PE ) was measured by flow cytometry for the BCR of interest (red = WT; blue = D368R). Grey is binding to an isotype control (for 122E binding) or binding to surface BCR negative (for surface BCR expression). (B) BCR binding to irrelevant Y98F influenza hemagglutinin (HA) probe that has been labeled with either PE or APC. Binding is to FE53 BCR, an HA-targeting BCR or to VRC01, Lin2 gHgL, or Lin2 sHsL (grey = binding to BCR-surface negative).

**A**

```
                                FR1                  CDR1      FR2                 CDR2                         FR3                             CDR3
               -------------------------++++++++-----------------++++++++-------------------------------------+++++++++++++++++++
IGVH1-2*02     QVQLVQSGAEVKKPGASVKVSCKASGYTFTGYYMHWVRQAPGQGLEWMGWINPNSGGTNYAQKFQGRVTMTRDTSISTAYMELSRLRSDDTAVYYCAR----------------
Lin2_gH        ***********************************************************************************************CARDGTSLLWFGESFYFDY
Lin2_sH        *********************************IL*********************H*************N*******************L*FCARDGTSLLWFGESFYFDY

                             FR1                  CDR1        FR2        CDR2                   FR3                  CDR3
                --------------------------++++++-----------------+++-----------------------------------+++++++++++
IGKV5-48*01     DILLTQSPAILSVSPGERVSFSCRASQSIGTSIHWYQQRTNGSPRLLIKYASESISGIPSRFSGSGSGTDFTLSINSVESEDIADYYC----------
Lin2_gL         ***************************************************************************************CQQSNSWPTLT
Lin2_sL         *******************************T*****************F******************************F****CQQSNSWPTLT
```

**B**

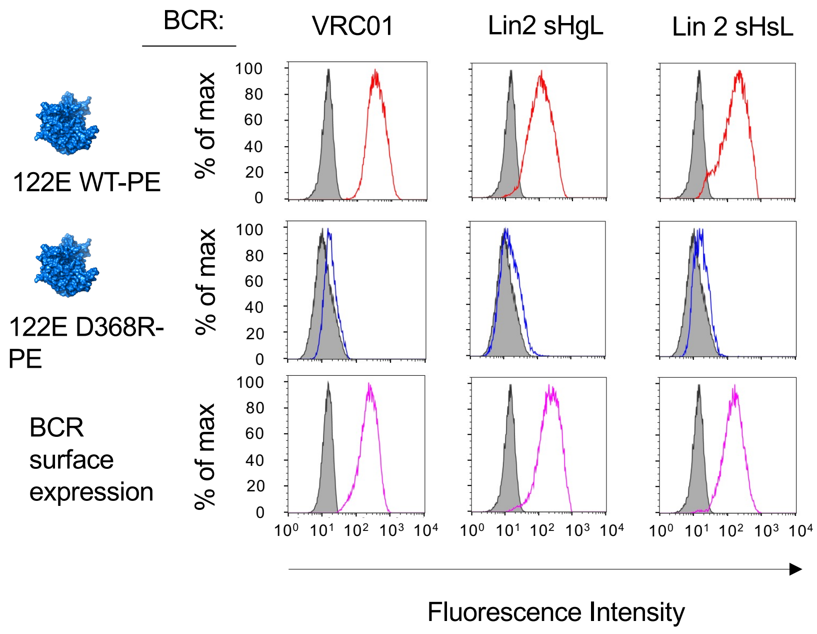


**Supplementary Figure 14. HC directed acquisition of D368R sensitivity by Lin2.** (A) gH, sH, gL, sL sequences of public clone Lin2. (B) 122E Env recognition by BCRs: VRC01; Lin2 sHgL; Lin2 sHsL. Binding with fluorescently labelled 122E (WT-PE vs D368R-APC) was measured by flow cytometry for the BCR of interest [red = WT; blue = D368R) and grey for isotype control (for 122E binding) or grey for surface BCR negative (for surface BCR expression)]. BCR surface expression was measured by anti-light chain reactivity (pink).

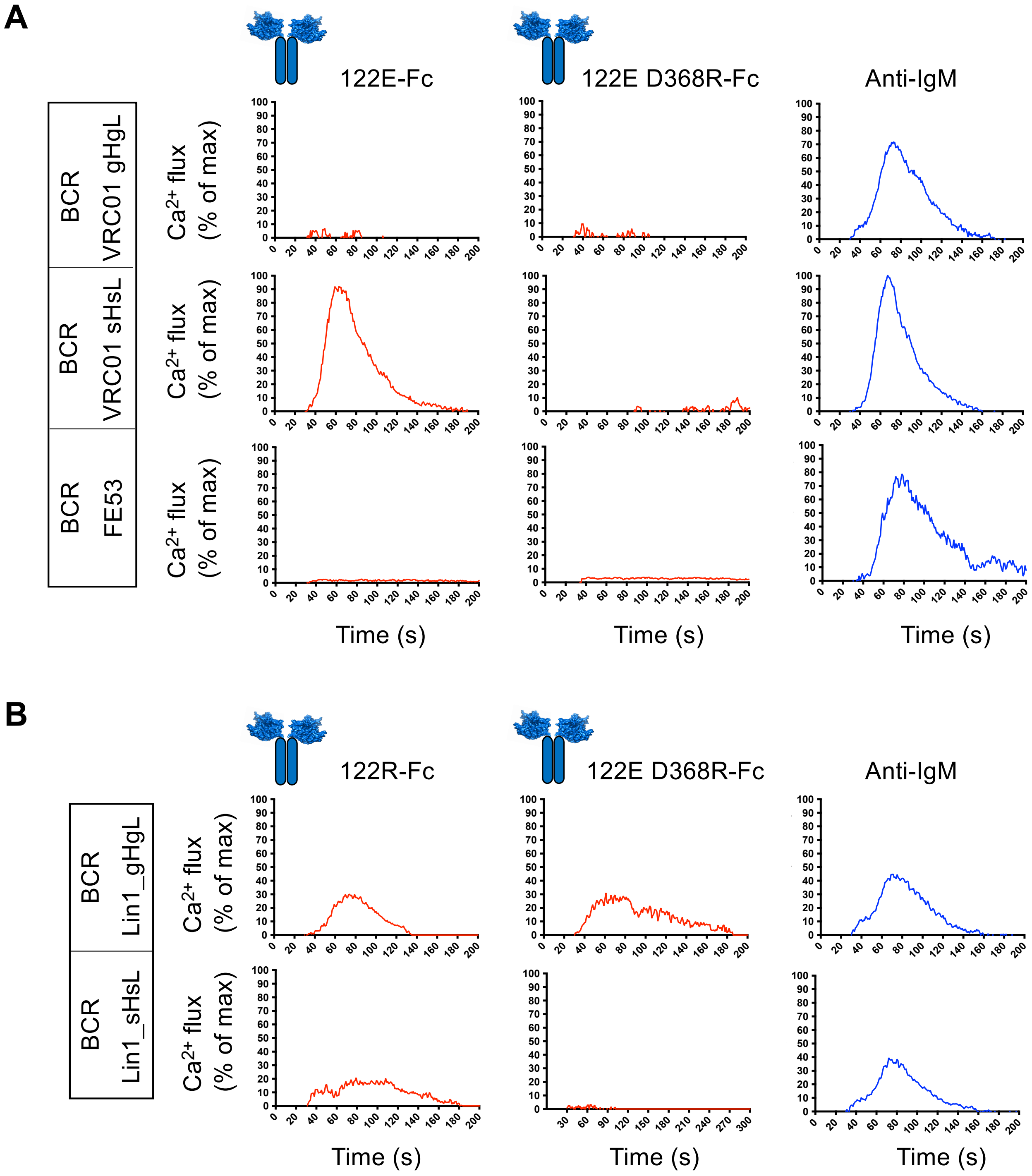


**Supplementary Figure 15. Isotype controls and Lin2 BCR triggering through 122E-Fc vs 122E D368R-Fc. (A)** $Ca^{2+}$ flux activity, measured kinetically on three B cell lines presenting IgM BCRs of known antigenicity: VRC01 gHgL (non-envelope specific); VRC01 (Env specific and D368R sensitive); and FE53 (specific for hemagglutinin from influenza virus). **(B)** Lin2 gHgL BCR vs Lin2 sHsL BCR triggering in response to bivalent 122E-Fc and/or 122E -D368R-Fc or anti-IgM. In all cases Fluxing was measured by the ratiometric $Ca^{2+}$ sensing dye fura red and normalized to total flux capacity, as defined by the ionophore ionomycin.

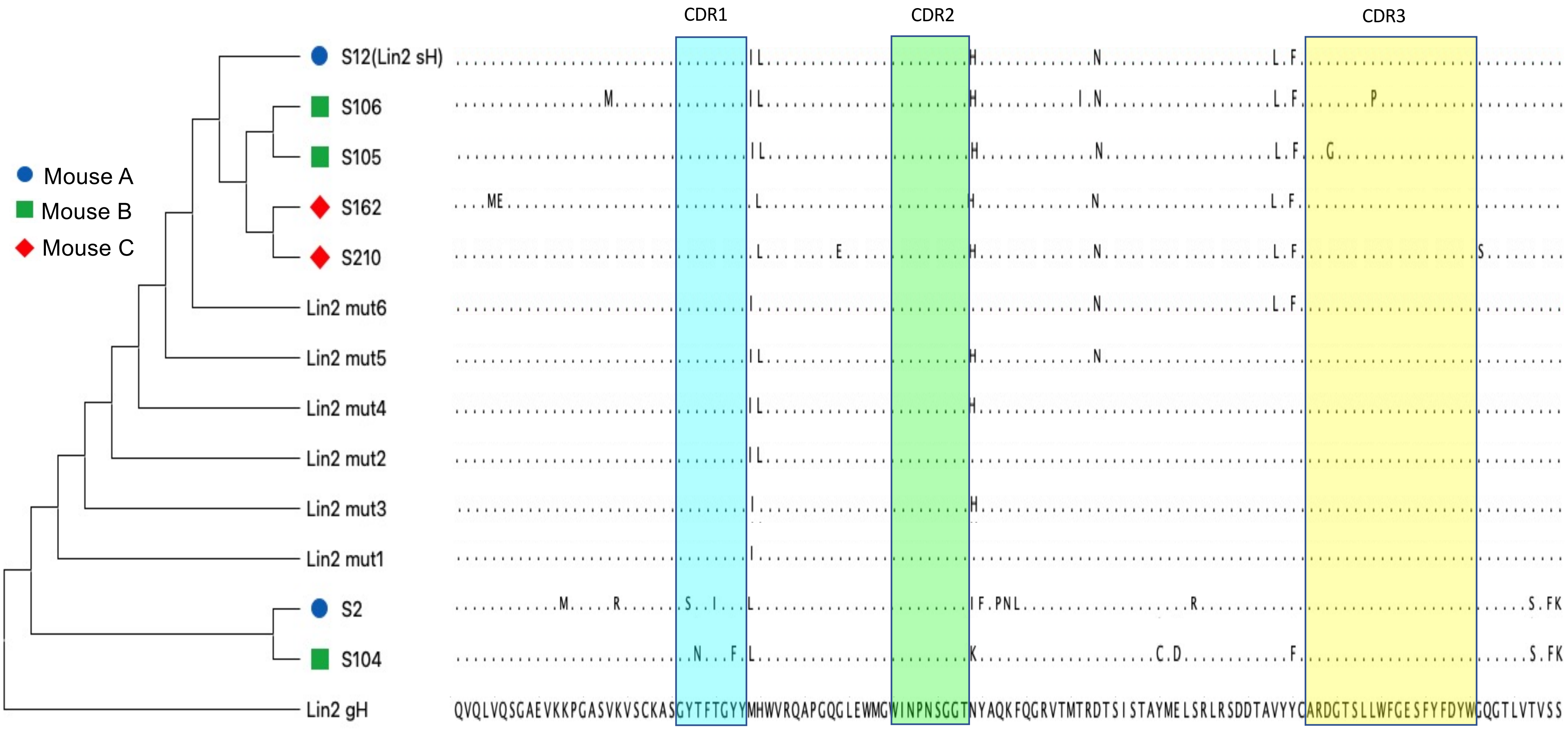


**Supplementary Figure 16. Phylogenic of Public Clonal Lineage 2 with inferred intermediates.** Public clonal lineages are defined as public CDRH3 + shared CDRL3 (see also Figure 2B and Supplementary Data 1). Phylogeny of Public Clonal Lineage 2 by HC nucleotide sequence. To broadly highlight where the inferred intermediates (Lin2_mut1-6) distribute within the public pathway, the tree was constructed using all the Public Lineage 2 clones from the three vaccine recipients.

**Supplementary Table 1** Binding constants for Fabs reconstituted from B cell lineages 1 and 2 (see also Figures 2B, 4C, 6C)

| Fab | Antigen (122e) | KD (M) | Ka ($M^{-1}$ $s^{-1}$) | Ka Error | Kd ($s^{-1}$) | Kd Error |
|---|---|---|---|---|---|---|
| Lin1_gHgL | WT | 1.56E-06 | 6.11E+04 | 2.85E+03 | 9.54E-02 | 2.31E-03 |
| | D368R | 1.75E-06 | 5.31E+04 | 2.56E+03 | 9.28E-02 | 2.39E-03 |
| Lin1_mut1 | WT | 5.79E-06 | 2.93E+04 | 9.45E+02 | 1.70E-01 | 1.76E-03 |
| | D368R | 5.31E-06 | 2.99E+04 | 8.55E+02 | 1.59E-01 | 1.72E-03 |
| Lin1_mut2 | WT | 2.98E-06 | 4.34E+04 | 9.09E+02 | 1.29E-01 | 1.11E-03 |
| | D368R | 4.01E-06 | 3.21E+04 | 8.92E+02 | 1.29E-01 | 1.41E-03 |
| Lin1_mut3 | WT | 4.39E-06 | 4.18E+04 | 1.14E+03 | 1.84E-01 | 1.92E-03 |
| | D368R | 4.47E-06 | 4.70E+04 | 1.42E+03 | 2.10E-01 | 2.30E-03 |
| Lin1_mut4 | WT | 1.01E-05 | 2.41E+04 | 1.05E+03 | 2.43E-01 | 3.11E-03 |
| | D368R | 1.36E-05 | 2.14E+04 | 1.37E+03 | 2.90E-01 | 4.54E-03 |
| Lin2_gHgL | WT | 8.55E-07 | 3.13E+04 | 3.97E+02 | 2.68E-02 | 3.13E-04 |
| | D368R | 8.08E-06 | 1.75E+04 | 8.15E+02 | 1.41E-01 | 2.38E-03 |
| Lin2_mut1 | WT | 1.17E-06 | 2.65E+04 | 4.29E+02 | 3.11E-02 | 3.40E-04 |
| | D368R | 7.00E-06 | 1.47E+04 | 4.11E+02 | 1.03E-01 | 1.03E-03 |
| Lin2_mut2 | WT | 4.25E-06 | 7.83E+03 | 9.94E+01 | 3.33E-02 | 2.42E-04 |
| | D368R | >100μM | 4.29E+01 | 7.38E+02 | 1.83E-01 | 4.16E-03 |
| Lin2_mut3 | WT | 4.82E-07 | 3.53E+04 | 4.53E+02 | 1.70E-02 | 2.03E-04 |
| | D368R | 1.62E-06 | 2.85E+04 | 2.89E+02 | 4.61E-02 | 2.98E-04 |
| Lin2_mut4 | WT | 2.44E-06 | 9.48E+03 | 8.37E+01 | 2.31E-02 | 1.39E-04 |
| | D368R | 1.79E-05 | 7.08E+03 | 3.55E+02 | 1.26E-01 | 1.38E-03 |
| Lin2_mut5 | WT | 2.90E-06 | 7.74E+03 | 6.22E+01 | 2.25E-02 | 1.14E-04 |
| | D368R | 2.39E-05 | 5.30E+03 | 3.98E+02 | 1.27E-01 | 1.70E-03 |
| Lin2_mut6 | WT | 1.60E-06 | 2.20E+04 | 2.61E+02 | 3.52E-02 | 2.80E-04 |
| | D368R | 9.92E-06 | 1.12E+04 | 3.53E+02 | 1.11E-01 | 1.06E-03 |
