# Supplementary material for: Engaging an HIV vaccine target through the acquisition of low B cell affinity
Source: Nat Commun. 2023 Aug 28;14:5249. doi: 10.1038/s41467-023-40918-2 (PMC10462694; doi:10.1038/s41467-023-40918-2)
Supplement: Supplementary file 2 — Description of Additional Supplementary Files [file 41467_2023_40918_MOESM2_ESM.pdf]

Description of Additional Supplementary Files

Supplementary Data 1

Sheet 1**:** BCR sequencing data for B cell lineages from mouse A, B, and C. Each BCR clone is annotated and designated in relation to a public use of CDRH3 (=public lineage) and public clonality (=shared CDRH3 + shared LC). This data is graphed in Figure 2A. The BCR VH, VL accession numbers are also provided to obtain the heavy chain and light chain nucleotide and amino acid sequences of each clone.

Sheet 2: CDRH3 length summary for the public B cell lineages from mouse A, B, and C.

Sheet 3: HIV Env amino acid sequences for 122E, 45B, 92C, YU2 along with their CD4bs mutant forms. The amino acid sequences for the dimeric Fc versions of these proteins used for BCR triggering are also provided.
