# Supplementary material for: Engaging an HIV vaccine target through the acquisition of low B cell affinity
Source: Nat Commun. 2023 Aug 28;14:5249. doi: 10.1038/s41467-023-40918-2 (PMC10462694; doi:10.1038/s41467-023-40918-2)
Supplement: Supplementary file 4 — Reporting Summary [file 41467_2023_40918_MOESM4_ESM.pdf]

nature portfolio

Corresponding author(s): Daniel Lingwood

Last updated by author(s): Jul 28, 2023

Reporting Summary

Nature Portfolio wishes to improve the reproducibility of the work that we publish. This form provides structure for consistency and transparency in reporting. For further information on Nature Portfolio policies, see our Editorial Policies and the Editorial Policy Checklist.

Statistics

For all statistical analyses, confirm that the following items are present in the figure legend, table legend, main text, or Methods section.

n/a	Confirmed	
☐	☒	The exact sample size (*n*) for each experimental group/condition, given as a discrete number and unit of measurement
☐	☒	A statement on whether measurements were taken from distinct samples or whether the same sample was measured repeatedly
☐	☒	The statistical test(s) used AND whether they are one- or two-sided<br>*Only common tests should be described solely by name; describe more complex techniques in the Methods section.*
☐	☒	A description of all covariates tested
☐	☒	A description of any assumptions or corrections, such as tests of normality and adjustment for multiple comparisons
☐	☒	A full description of the statistical parameters including central tendency (e.g. means) or other basic estimates (e.g. regression coefficient) AND variation (e.g. standard deviation) or associated estimates of uncertainty (e.g. confidence intervals)
☐	☒	For null hypothesis testing, the test statistic (e.g. *F*, *t*, *r*) with confidence intervals, effect sizes, degrees of freedom and *P* value noted<br>*Give P values as exact values whenever suitable.*
☒	☐	For Bayesian analysis, information on the choice of priors and Markov chain Monte Carlo settings
☐	☐	For hierarchical and complex designs, identification of the appropriate level for tests and full reporting of outcomes
☒	☐	Estimates of effect sizes (e.g. Cohen's *d*, Pearson's *r*), indicating how they were calculated

Our web collection on statistics for biologists contains articles on many of the points above.

Software and code

Policy information about availability of computer code

Data collection	no code was used in this data collection
Data analysis	Microsoft Excel, Prism 9.1.0, FlowJo 10.7.2, Cloanalyst (https://www.bu.edu/computationalimmunology/research/software/)

For manuscripts utilizing custom algorithms or software that are central to the research but not yet described in published literature, software must be made available to editors and reviewers. We strongly encourage code deposition in a community repository (e.g. GitHub). See the Nature Portfolio guidelines for submitting code & software for further information.

Data

Policy information about availability of data

All manuscripts must include a data availability statement. This statement should provide the following information, where applicable:

- Accession codes, unique identifiers, or web links for publicly available datasets
- A description of any restrictions on data availability
- For clinical datasets or third party data, please ensure that the statement adheres to our policy

Data used in this paper is made fully available in Supplementary Data 1 and the Data Source file. Antibody sequences are deposited in GenBank and their accession numbers are listed in Supplementary Data 1.

Research involving human participants, their data, or biological material

Policy information about studies with human participants or human data. See also policy information about sex, gender (identity/presentation), and sexual orientation and race, ethnicity and racism.

Reporting on sex and gender	N/A
Reporting on race, ethnicity, or other socially relevant groupings	N/A
Population characteristics	N/A
Recruitment	N/A
Ethics oversight	N/A

Note that full information on the approval of the study protocol must also be provided in the manuscript.

Field-specific reporting

Please select the one below that is the best fit for your research. If you are not sure, read the appropriate sections before making your selection.

[x] Life sciences [ ] Behavioural & social sciences [ ] Ecological, evolutionary & environmental sciences

For a reference copy of the document with all sections, see nature.com/documents/nr-reporting-summary-flat.pdf

Life sciences study design

All studies must disclose on these points even when the disclosure is negative.

Sample size	Power calculations were performed using online software (https://sample-size.net) to determine sample sizes. Given our prior experience with these animal models, we have previously observed an effect size of ~2.4. Given a need for 80% power to detect an alpha level of 0.05, we require a minimum of n=4 animals per experimental group at this effect size. Accordingly our experiments deployed either n=4 or n=5 animals per experimental group.
Data exclusions	No data was excluded.
Replication	Experiments were independently replicated to insure reproducibility and also analyzed by orthogonal methods (e.g. serum antibody titer vs antigen-specific B cell memory) and the details of this are noted in the figure legends. These attempts at replication were successful.
Randomization	Male and female mice were obtained from breeding and assigned a number with a random number generator (https://www.random.org) and treatment groups were assigned according to absolute values (e.g top 5 random numbers = treatment 1, bottom 5 random numbers = treatment 2)
Blinding	Blinding was not possible because the same person often generated the recombinant reagents (vaccine immunogens, and BCR triggering ligands) used for downstream experimentation.

Reporting for specific materials, systems and methods

We require information from authors about some types of materials, experimental systems and methods used in many studies. Here, indicate whether each material, system or method listed is relevant to your study. If you are not sure if a list item applies to your research, read the appropriate section before selecting a response.

Materials & experimental systems

n/a	Involved in the study	
[ ]	[x]	Antibodies
[ ]	[x]	Eukaryotic cell lines
[x]	[ ]	Palaeontology and archaeology
[ ]	[x]	Animals and other organisms
[x]	[ ]	Clinical data
[x]	[ ]	Dual use research of concern
[x]	[ ]	Plants

Methods

n/a	Involved in the study	
[x]	[ ]	ChIP-seq
[ ]	[x]	Flow cytometry
[x]	[ ]	MRI-based neuroimaging

Antibodies

Antibodies used	Brilliant Violet 421™ anti-mouse CD19 Antibody, BioLegend (Cat No 115537) (final dilution = 1 in 100)<br>Brilliant Violet 605™ anti-mouse IgM Antibody, BioLegend (Cat No 406523) (final dilution = 1 in 100)<br>PerCP/Cyanine5.5 Goat anti-mouse IgG Antibody, BioLegend (Cat No 405314) (final dilution = 1 in 100)<br>Brilliant Violet 785™ anti-mouse CD3 Antibody (Cat No 100232) (final dilution = 1 in 100)<br>BUV395 Rat Anti-Mouse IgD, BD Biosciences (Cat no: 564274) (final dilution = 1 in 100)<br>PE/Cyanine7 anti-mouse GL7 Antibody, BioLegend (Cat no: 144620) (final dilution = 1 in 100)<br>Alexa Fluor® 594 anti-mouse CD38 Antibody, BioLegend (Cat no: 102725) (final dilution = 1 in 100)
Validation	Brilliant Violet 421™ anti-mouse CD19 Antibody, BioLegend (Cat No 115537): Manufacturer validation: quality control tested by flow cytometry and immunohisitochemistry. 56 references.<br><br>Brilliant Violet 605™ anti-mouse IgM Antibody, BioLegend (Cat No 406523): Manufacturer validation: quality control tested by flow cytometry. 9 references.<br><br>PerCP/Cyanine5.5 Goat anti-mouse IgG (minimal x-reactivity) Antibody, BioLegend (Cat No 405314): Manufacturer validation: quality control tested by flow cytometry. 7 references.<br><br>Brilliant Violet 785™ anti-mouse CD3 Antibody, BioLegend (Cat No 100232): Manufacturer validation: quality control tested by flow cytometry. 32 references.<br><br>BUV395 Rat Anti-Mouse IgD, BD Biosciences (Cat no: 564274): Manufacturer validation: routine testing by flow cytometry. 4 references.<br><br>PE/Cyanine7 anti-mouse GL7 Antibody, BioLegend (Cat no: 144620): Manufacturer validation: quality control tested by flow cytometry. 10 references.<br><br>Alexa Fluor® 594 anti-mouse CD38 Antibody, BioLegend (Cat no: 102725). Manufacturer validation: quality control tested immunohisitochemistry. 4 references

Eukaryotic cell lines

Policy information about cell lines and Sex and Gender in Research

Cell line source(s)	FreeStyle™ 293-F Cells (Thermofisher, R79007), Ramos B cell IgM surface negative B cell line [Ramos B cells from ATCC (CRL-1596) and IgM negative version from Weaver et al. 2016, PMID 26741406)
Authentication	FreeStyle™ 293-F Cells: morphology under cell counter. Cells are direct from Thermo.<br><br>Ramos B cell IgM surface negative B cell line expressing Lin1 and Lin2 BCR variants: flow cytometry and plasmid sequencing
Mycoplasma contamination	All cell lines are negative for mycoplasma
Commonly misidentified lines<br>(See ICLAC register)	N/A

Animals and other research organisms

Policy information about studies involving animals; ARRIVE guidelines recommended for reporting animal research, and Sex and Gender in Research

Laboratory animals	Wildtype C57Bl/6 mice and transgenic mice on C57Bl/6 background (stain previously described in PMID: 31563464)
Wild animals	No wild animals were used
Reporting on sex	Both female and males were obtained by breeding and deployed as experimental animals. Experimental groups were between n=4 and n=5 and data disaggregated for sex is available in the Source Data file. Sex-based differences were not observed either in relation to responsiveness to antigen and we found that the same public B cell response enabling permissive BCR scanning of the Env CD4bs was present in both males and female mice.
Field-collected samples	No field collected samples were used
Ethics oversight	Institutional Animal Care and Use Committee (IACUC), Massachusetts General Hospital. (Approved IACUC protocol #2014N000252)

Note that full information on the approval of the study protocol must also be provided in the manuscript.

Flow Cytometry

Plots

Confirm that:

- [x] The axis labels state the marker and fluorochrome used (e.g. CD4-FITC).
- [x] The axis scales are clearly visible. Include numbers along axes only for bottom left plot of group (a 'group' is an analysis of identical markers).
- [x] All plots are contour plots with outliers or pseudocolor plots.
- [x] A numerical value for number of cells or percentage (with statistics) is provided.

Methodology

Sample preparation	Mouse spleens were gently ground in PBS, lysed in 1xACK lysis buffer and filtered through a 70µm cell strainer. The cells were washed (PBS) and then stained with Aqua Live/Dead amine-reactive dye and calcein AM (0.025 mg/ml for 2 minutes). The cells were then washed and then incubated with a 1x cocktail of flow cytometry antibodies: anti-CD3 Brilliant Violet 785; anti-CD19 BV421; anti-IgM BV605; anti-IgD BUV395; anti-IgG PerCPCy5.5; anti-GL7 PE-Cy7; anti-CD38 Alexa 594; along with 0.25µg of Env-PE, Env-APC-Cy7 and Env-D368R-APC probes The mixture was incubated at 4°C for 1 hour. The cells were then washed twice, resuspended in PBS and then subjected to flow cytometry.
Instrument	FACS Aria Fusion Sorter (BD Biosciences)
Software	BD FACSDiva, Flowjo
Cell population abundance	Representative gating for flow sort:<br>All events: 2,000,000<br>FSC/SSC gate: 1178345<br>Live: 1149801<br>B cell (CD19+/CD3-): 327,941<br>IgG+/IgM-/IgD-: 12058<br>IgG+/IgM-/IgD/GL7-/CD38+/Env+/Env-D368R+ : 341<br>IgG+/gM-/IgD/GL7-/CD38+/Env+/Env-D368R-: 150
Gating strategy	The vaccine-expanded memory IgG B cells specific for the CD4bs on Env were defined as CD3-/CD19+/IgM-/IgD-/IgG+/GL7-/CD38+/Env+/Env-D368R-

- [x] Tick this box to confirm that a figure exemplifying the gating strategy is provided in the Supplementary Information.
